# Melatonin Enhances Seed Germination and Seedling Growth of *Medicago sativa* Under Salinity *via* a Putative Melatonin Receptor *MsPMTR1*

**DOI:** 10.3389/fpls.2021.702875

**Published:** 2021-08-17

**Authors:** Ruonan Yu, Tiantian Zuo, Pengfei Diao, Jiabin Fu, Yanyan Fan, Yue Wang, Qiqi Zhao, Xuesong Ma, Wenting Lu, Aoga Li, Ru Wang, Fang Yan, Li Pu, Yiding Niu, Hada Wuriyanghan

**Affiliations:** ^1^Key Laboratory of Forage and Endemic Crop Biotechnology, Ministry of Education, School of Life Sciences, Inner Mongolia University, Hohhot, China; ^2^Department of Medicine, Ordos Institute of Technology, Ordos, China; ^3^Biotechnology Research Institute, Chinese Academy of Agricultural Sciences, Beijing, China

**Keywords:** *Medicago sativa*, salinity, melatonin, physiological mechanism, transcriptional responses, *MsPMTR1*, seed germination, plant growth

## Abstract

Alfalfa (*Medicago sativa* L.) is an important forage crop, and salt stress is a major limiting factor in its yield. Melatonin (MT) is a multi-regulatory molecule in plants. We showed that basal MT content was positively correlated with the salt tolerance degree of different alfalfa varieties. MT and its precursor 5-HT fully recovered seed germination while partially ameliorated seedling growth of salt-stressed alfalfa. The 5-HT showed some divergent effects from MT with regards to growth amelioration under salinity. Salt stress caused stunted plant growth in soil culture, while MT ameliorated it by elevating plant height, fresh weight, branching number, and chlorophyll content. Silencing of a putative MT receptor, *MsPMTR1*, which was shown to be membrane-localized, abolished the ameliorative effects of MT on salt-stressed alfalfa seedling growth, while overexpression of *MsPMTR1* improved plant growth under salt stress. The RNA sequencing analysis showed that nine pathway genes were specifically induced by MT treatment compared with salt stress. These MT-responsive differentially expressed genes include basal metabolic pathway genes, such as “ribosome, elongation factor,” “sugar and lipid metabolism,” and “photosynthesis” and stress-related genes encoding “membrane integrity” related proteins, heat shock protein, peroxidase/oxidoreductase, and protease. Several abiotic stress response-related genes, such as *DRE*, *ARF*, *HD-ZF*, *MYB*, and *REM* were repressed by NaCl treatment while induced by MT treatment. In summary, we demonstrated the importance of *MsPMTR1* in MT-mediated salt tolerance in alfalfa, and we also analyzed the regulatory mechanism of MT during alfalfa seed germination under salt stress.

## Introduction

Soil salinization is one of the most serious environmental stresses to crop production. Salt stress influences around 20% of the cultivated lands and nearly 50% of all irrigated lands worldwide (Zhu, 2001). Salinity affects plant growth and development through osmotic, ionic, and oxidative stresses. Seed germination is the critical stage in the whole life cycle of plants, and it is an extremely vulnerable stage to salt damage (Ibrahim, 2016). Salt stress also causes morphological impairments and hence also retards vegetative growth. While low or moderate salt concentration affects plant growth *via* osmotic stress, the high salt concentration would render the plant to ionic stress. The excessive Na^+^ accumulation also leads to membrane injury by oxidative stress (Isayenkov and Maathuis, 2019).

Plants evolved sophisticated molecular and physiological strategies to cope with salt stress. Under salt stress, plants can initiate osmoregulatory mechanism by accumulating low-molecular-weight osmolytes, such as glycine, betaine, proline, and other amino acids, free sugars, and polyols (Munns et al., 2020). The ability of plants to maintain a high cytosolic K^+^/Na^+^ ratio under ion toxicity is another salt tolerance mechanism (Zhu, 2003). Plant stress responses depend on many factors, among which plant hormones are key endogenous molecules that play important roles in improving stress tolerances (Julkowska and Testerink, 2015; Verma et al., 2016). Salt stress causes oxidative damages by the accumulation of reactive oxygen species (ROS), which includes hydroxyl radical (OH^–^), superoxide anion (O^2–^), and hydrogen peroxide (H_2_O_2_). Antioxidant protection systems play important roles in protecting membrane structure by removing excessive ROS. The ROS scavenging enzymatic system includes peroxidase (POD), catalase (CAT), superoxide dismutase (SOD), ascorbate peroxidase (APX), and glutathione reductase (GR) (Yang and Guo, 2018). Nonenzymatic ROS scavenging activity attributes to the coordinated accumulation of alkaloids, carotenoids, tocopherol, ascorbic acid (ASA), and glutathione (GSH) (Noctor and Foyer, 1998).

Melatonin (MT, *N*-acetyl-5-methoxytryptamine) is a kind of indoleamine substance that has pleiotropic biological functions in both animals and plants (Dubbels et al., 1995; Hattori et al., 1995; Murch et al., 1997). Although identified first in animals (Reiter, 1991), the MT was isolated in Japanese morning glory in 1995 and then was detected in many higher plants (Tassel and O’Neill, 2001). The MT has an efficacious ROS and reactive nitrogen species (RNS) scavenging activity and protects biomolecules from oxidative stress (García et al., 2014; Tan et al., 2015; Khan et al., 2020). The MT reacts with hydroxyl and peroxy radicals to directly scavenge H_2_O_2_ and OH^–^ (Galano et al., 2011; Reiter et al., 2016). Furthermore, MT also indirectly regulates free radical scavenging by increasing the activities of antioxidant enzymes, such as SOD, CAT, POD, and glutathione peroxidase (GPX) (Arnao and Hernández-Ruiz, 2018). The MT could regulate seed germination (Zhang et al., 2014b), root growth (Chen et al., 2009), photosynthesis (Zhang et al., 2019), and fruit ripening (Pérez-Llorca et al., 2019). The MT also alleviates the damage caused by environmental stresses, such as cold, drought, salinity, heavy metals, alkali ions, ultraviolet radiation, as well as biotic stresses on higher plants (Wang et al., 2017). Especially, MT was reported to ameliorate salt stress in higher plants (Ke et al., 2018; Michard and Simon, 2020). At present, the biosynthetic pathway of MT has been unveiled in several model plant species (Posmyk and Janas, 2009; Nawaz et al., 2016). Tryptophan-5-hydroxylase (T5H) is a key enzyme and serotonin (5-hydroxytryptamine, 5-HT) is an important intermediate for MT biosynthesis (Zuo et al., 2014). The MT is universally present in many plant species with greatly varying contents (Nawaz et al., 2016). A variety of stress conditions were reported to induce endogenous MT accumulation, which was associated with the alleviation of the stress damage. Exogenous application of MT or overexpression of MT biosynthesis genes can also improve the ability of plants to cope with adversity (Yin et al., 2013).

Alfalfa (*Medicago sativa* L.) is the most widely cultivated perennial legume forage in many countries. Because of its high protein content and excellent palatability for livestock, alfalfa is known as the “king of herbage.” Alfalfa is originated in central Asia and it is suitable for growing in neutral or moderate saline-alkaline soil, while high salinity presents threats to its yield (Singer et al., 2018). In this study, four varieties of alfalfa with different salt tolerance (Bingchi, Golden Empress, Weiner, and Algonquin) were used as materials to study the effect of exogenous MT on seed germination and seedling growth of alfalfa under NaCl stress. We have also investigated the effects of MT on osmotic regulation, ion regulation, and antioxidant system of alfalfa seedlings under NaCl stress. Salt-sensitive Bingchi variety was used for pot experiment and functional elucidation of a putative MT receptor gene *MsPMTR1* from alfalfa. The transcriptional regulatory mechanism of salt stress and MT treatment was uncovered by RNA-seq and the differentially expressed genes (DEGs) analysis.

## Materials and Methods

### Germination Test and Measurement of Growth and Physiological Parameters

The experiments were carried out according to our previous publication (Wang et al., 2020). Briefly, seeds from different alfalfa varieties (Bingchi, Algonquin, Golden Empress, and Weiner) were sterilized, and one hundred seeds were used for germination test at 2°C in a growth incubator. The photoperiod was 16 h light and 8 h dark, and the luminous flux density was 40 μmol/m^2^ s. Different chemicals, including NaCl, MT, and 5-HT were applied separately or together. Germination rate, fresh weight, dry weight, shoot length, root length, and relative water content were measured on the 10th day post-treatment. For combined treatment, seeds were exposed to various combinations of solutions with 150 μM MT and 1 μM 5-HT together with 250 mM NaCl, respectively. On the 7th day, the root tissue was used for the measurement of Na^+^ content, K^+^/Na^+^ ratio, MT content, PRO content, malondialdehyde (MDA) content, H_2_O_2_ content, and POD, SOD, CAT activities by different detection kits (Suzhou Keming Biotechnology Co., Ltd, Jiangsu, China) according to the instructions of the manufacturer. The fresh plants were washed three times with ddH_2_O, and a 0.1 g sample was used as the experimental material. Diaminobenzidine (DAB) staining was performed at the same time. Seedling was soaked in 1 mg/ml DAB solution (50 mM *Tris*–HCl, pH = 4.0). After vacuum infiltration and decoloration with absolute alcohol, the seedling was photographed.

### RNA-Seq and Analysis of DEGs

Seeds from alfalfa variety, Bingchi were germinated for 7 days under treatment of 250 mM NaCl alone; 250 mM NaCl + 150 μM MT (Kang et al., 2010), or control water RNA-seq was performed according to the literature (Zhao et al., 2018). Root tissue of three biological replicates, which were individually collected from each treatment, were used for total RNA isolation, library preparation, transcriptome sequencing on Illumina Hiseq platform, and paired-end reads were generated. Raw data were deposited in Sequence Read Archive (SRA)^1^ database with the number PRJNA742658. Raw reads were processed through in-house Perl scripts to obtain clean reads. Differential expression analysis of unigenes between different treatment groups was performed using the DESeq R package (1.10.1). The *t*-test analysis on DEGs was carried out between different treatments, and a heatmap was drawn by MeV software. The expression of twenty selected unigenes was confirmed by RT-qPCR experiments using gene-specific primers listed in Supplementary Table 1.

### Pot Culture Experiment and Determination of Growth Parameters

Seeds of alfalfa variety, Bingchi were germinated for 1 week in sterile water, and the seedlings were transplanted into the pot containing the nutrient soil (Peat and perlite supplemented with N, K, P, Ca, and Mn): vermiculite (1:1). Seedlings were grown in a greenhouse at 24°C with a photoperiod of 16h:8h (light: dark) and relative humidity of 70%. After the seedlings were grown for 1 week, they were treated with 137 mM NaCl, which was selected according to the literature (Zhang et al., 2014a; Wang et al., 2020). Briefly, the soil was saturated with 137 mM NaCl solution and replaced two times with water solution, and the alternate treatment of NaCl and water solution was repeated. At 14 dpi, the seedlings were irrigated with 150 μM MT or 1 μM 5-H Tat dark condition, respectively, with NaCl. On 30th day post-treatments, representative individuals from each group were photographed. At least 20 individuals were measured for plant height, tiller number, branch number, fresh weight, dry weight, and relative water content. Around 50 leaves were randomly selected from each treatment, and the relative content of chlorophyll in leaves was detected by a hand-held chlorophyll analyzer (REnQ-VI, SPAD-502 PLUS, KONICA MINOLTA, Japan).

### Recombinant Vector Construction for *MsPMTR1* and Subcellular Localization

A putative melatonin receptor gene, *MsPMTR1* was identified from transcriptome data of *M*. *sativa via* homology search using the *Arabidopsis thaliana AtCAND2/PMRT1* sequence. Interference sequence for *MsPMTR1* was cloned from alfalfa variety, Bingchi cDNA with primers (forward: 5′-G*GAATTC* AAGAACTGGAACATTCTGGAAATGGTTGAAAAATCGGTA CTT-3′ and reverse: 5′-CG*GGATCC*ACTTCAAGCGAGGAG TACCAT-3′). Italics indicate *EcoR*I or *BamH*I restriction sites, and the underlined sequence indicates *MtTAS3a* sequence for tasiRNA induced silencing. The *MsPMTR1* interference sequence was ligated into the entry vector, pENTR with *EcoR*I/*BamH*I, and was subcloned into the binary vector, pKGWRR-RFP using Gateway recombination technology to obtain the RNAi recombinant vector, pKGWRR-*MsPMTR1*. For *MsPMTR1* overexpression, *MsPMTR1* gene ORF sequence was amplified by the primers 5′-ATGGTTGAAAAATCGGTACTTTCAA-3′ and 5′-CTCCCAGTCTGATTCGAAAAAACCA-3′, and was ligated into pCambia1307 vector. For subcellular localization of *MsPMTR1*, the same *MsPMTR1* gene ORF sequence was ligated into the pEarleyGate103-SL vector. The recombinant vector was transformed to *Agrobacterium tumefaciens* strain GV3101, infiltrated into *N*. *benthamiana* leaves, and GFP signal (excitation at 488 nm) was visualized at 36 hpi. Plasma membrane marker protein, PTIG6 fused with mCherry (excitation at 580 nm) was used in the same experiment.

### Development of *MsPMTR1* Overexpression and RNAi Alfalfa Lines

Thelfalfa variety, Bingchi was transformed with pKGWRR-*MsPMTR1 via* hairy root transformation according to the literature (Wang et al., 2020). Briefly, seeds were sterilized and evenly spread in 0.8% of Flame Spectroscopy (FS)-sterilized solid medium. Root tip was cut off with a sterile knife by about 3 mm and soaked with the *Agrobacterium* solution at the wound site and put in a 1.5% of FS-sterilized solid medium. The seedlings were grown at a greenhouse for about 3 weeks. Transgenic seedlings were confirmed by the detection of RFP signals under a stereo microscope (Nikon, SMZ18). The positive seedlings were then confirmed by the RT-qPCR experiment for *MsPMTR1* knockdown. *MsPMTR1* specific primers, 5′-TAACAAAGGGATTCACCGAT-3′ and 5′-AATTATGATATAAGAGCGACCA-3′, were used for amplification of *MsPMTR1* mRNA. Quantitative PCR (qPCR) reaction was performed in TransStart^TM^ Tip Green qPCR SuperMix on qTOWER 2.2 (Analytikjena) platform. The mRNA level of *M*. *sativa β-actin* (Accession number *JQ028730*.*1*) gene was used as an internal control by primers, 5′-CCGACCTCGTCATACTGGTG-3′ and 5′-TCTTCAGGAGCAACACGCAA-3′. Relative expression of target *MsPMTR1* gene was calculated by 2^–ΔΔ*CT*^ method. The seedlings were transferred to a soil pot with perlite and vermiculite (1:1) in a greenhouse (25°C, 16 h light / 22°C, 8 h dark) for 1 week. The seedlings were irrigated with 137 mM NaCl or together with 150 μM MT. On the 10th day post-treatments, representative individuals from each group were photographed. At least 20 individuals were measured for plant height, tiller number, number of leaves, and mortality rates. The *MsPMTR1* overexpression lines were obtained by the transformation of pCambia1307-*MsPMTR1* into *M*. *truncatula* cultivar R108 using the leaf explant method according to the literature (Cosson et al., 2006). Transgenic lines were confirmed by genomic DNA PCR using 35S primers, and expression of *MsPMTR1* was detected by RT-PCR analysis *via MsPMTR1* specific primers. T_1_ transgenic plants were used for NaCl treatment and phenotypic analysis.

### Accession Numbers

*Arabidopsis thaliana* AtCAND2/PMRT1 sequence (Accession No. OAP02387.1) and alfalfa *MsActin* (Accession No. JQ028730.1) sequences were obtained from the NCBI database. A putative melatonin receptor gene, *MsPMTR1* (Unigene ID number: Cluster-15808.11987) was identified from transcriptome data of *M*. *sativa via* homology search using the *A*. *thaliana* AtCAND2/PMRT1 sequence.

### Statistical Analysis

All experiments included at least three biological repeats, each expressed as mean ± SE. The Excel 2010 software was used to analyze the data, and statistical variance was analyzed with SPSS 19.0 software. The data for each variety under different treatment conditions were randomly assigned and independently calculated. The consistency and variance of data among each group were evaluated with SPSS 19.0 software. Based on this, one-way ANOVA was used for the analyses of between-group differences.

## Results

### Salt Stress Inhibits Seed Germination and Seedling Growth of Different Alfalfa Cultivars

**FIGURE 1:**
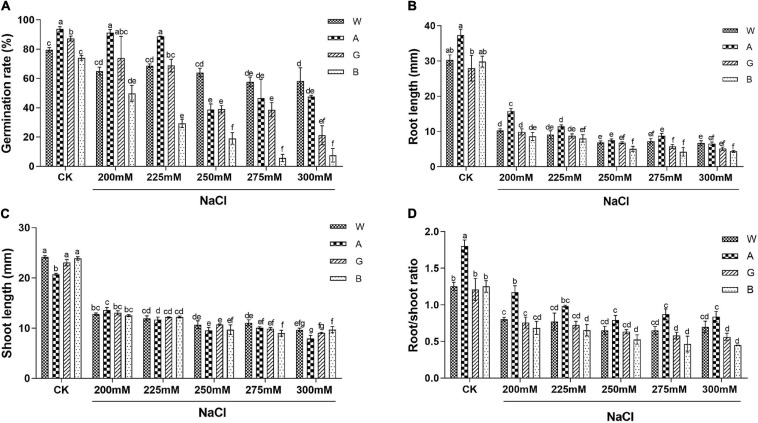
Salt stress inhibited germination and the growth of different alfalfa varieties. CK: Water. NaCl: 200, 225, 250, 275, and 300 mM. For each replicate, one hundred alfalfa seeds were surface-sterilized, spread evenly on two layers of filter paper which were presoaked with 5 ml treatment solution, and placed in a Petri dish at 20°C in an illuminating incubator. **(A)** Germination rate. Germinated seeds were calculated on the 10th day and germination rates were determined. Four biological replicates were performed and error bars show the SE values. **(B)** Root length. **(C)** Shoot length. **(D)** Root/shoot ratio. *n* = 20–40. The *t*-test was performed between samples in different treatment groups. Different numbers indicated significant differences (*p* ≦ 0.05) between different treatments. A, Algonquin; B, Bingchi; G, Golden Empress; W, Weiner.

When germinating seeds of four alfalfa varieties (Bingchi, Golden Empress, Weiner, and Algonquin) were salt-treated, germination rates were reduced in a dose-dependent manner. Bingchi was the most sensitive variety which had the lowest germination rate (8% at 275 mM NaCl) under each NaCl concentration. Weiner was the tolerant one where germination rate was significantly decreased by 27% at as higher as 275 mM of NaCl treatment, while Algonquin and Golden Empress were medium-tolerant varieties with germination rate decreases by 59 and 55% at 250 mM NaCl, respectively (Figure 1A and Supplementary Figure 1). Based on the above results, the degree of salt tolerance of the four varieties was determined as Weiner > Algonquin ≧ Golden Empress > Bingchi.

Several growth-related parameters besides seed germination were also measured. Salt stress significantly inhibited the growth of alfalfa seedlings. Even under the low salt concentration (200 mM), the root and shoot growth of the four varieties were significantly retarded by 58–71 and 34–43%, respectively, compared with the control group (Figures 1B,C). Root growth was more severely inhibited by salt stress than shoot growth (Figure 1D). Consistent with the retardation in seedling growth, the fresh weight of alfalfa seedling decreased significantly under as lower as 200 mM NaCl (Supplementary Figure 2A). On the contrary, salt stress had a little negative effect on the dry weight of alfalfa seedlings (Supplementary Figure 2B). The relative water contents of the four alfalfa varieties were 91–93% at the unstressed condition and were decreased to 77–80% after 300 mM NaCl treatment, indicating that the decrease in fresh weight was mainly due to the inhibition of cell water absorption by salt stress (Supplementary Figure 2C).

### Melatonin Ameliorates Salt Stress Inhibition on Alfalfa Seedlings

**FIGURE 2:**
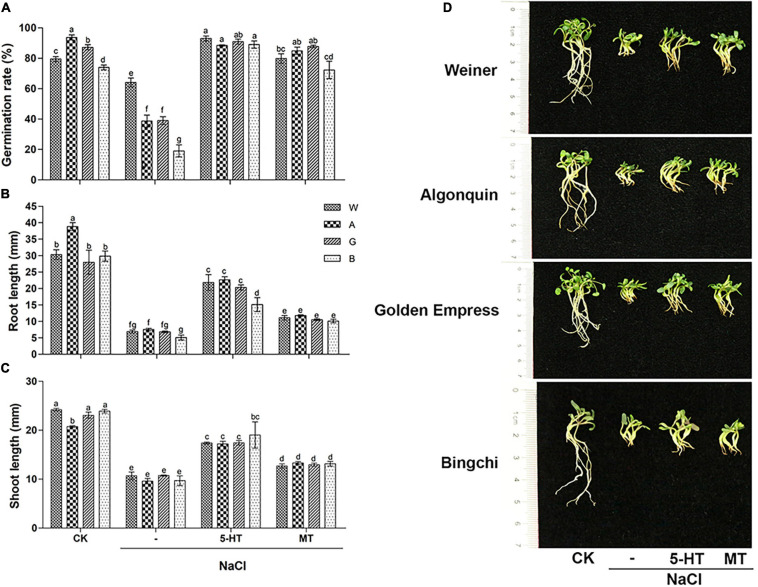
Effects of melatonin (MT) and 5-HT treatment on seed germination and seedling growths of different alfalfa varieties under salt stress. CK: Water. NaCl: 250 mM. MT: 150 μM. 5-HT: 1 μM. Seeds were germinated for 10 days under different treatments, and germination rate, *n* = 200–500 **(A)**, root length, *n* = 30–40 **(B)**, shoot length, *n* = 30–40 **(C)**, and seedling phenotype **(D)** were shown. Error bars show the SE between biological replicates and a *t*-test was performed between different treatment groups. Different numbers indicated significant differences (*p* ≦ 0.05) between samples. Representative seedlings were photographed and a scale bar was shown on the left. A, Algonquin; B, Bingchi; G, Golden Empress; W, Weiner.

To examine the role of MT, salt-stressed alfalfa seeds were treated with exogenous MT and 5-HT, an MT biosynthesis precursor. The optimal concentration for MT and 5-HT were determined in initial experiments (Supplementary Figures 3, 4). Both MT and 5-HT fully abolished the inhibition of salt stress on seed germination by recovering germination rates to control NaCl-free levels (Figure 2A). Compared to its complete ameliorative effects on germination rate, MT treatment slightly recovered seedling growth by elevating the root and shoot length by 38–50 and 16–28%, respectively, compared with NaCl treatment (Figures 2B,C). Salt stress reduced the fresh weight and relative water content of alfalfa seedlings, while MT treatment increased both of them by 19–35 and 3.1–4.7%, respectively. Compared with MT, 5-HT showed stronger alleviating effects on seedling growth, elevating the root length (66–68%), the shoot length (38–49%), fresh weight (45–49%), and relative water content (3.9–5.1%) to a higher level than that by MT treatment. On the contrary, no change in dry weight was observed under both MT and 5-HT treatments compared with NaCl treatment (Figure 2 and Supplementary Figures 5A–C). The data demonstrate that MT and 5-HT mainly influence water absorption but not biomass at this growth stage. The phenotypic observation is consistent with the above measurement on the shoot and root length (Figure 2D).

**FIGURE 3:**
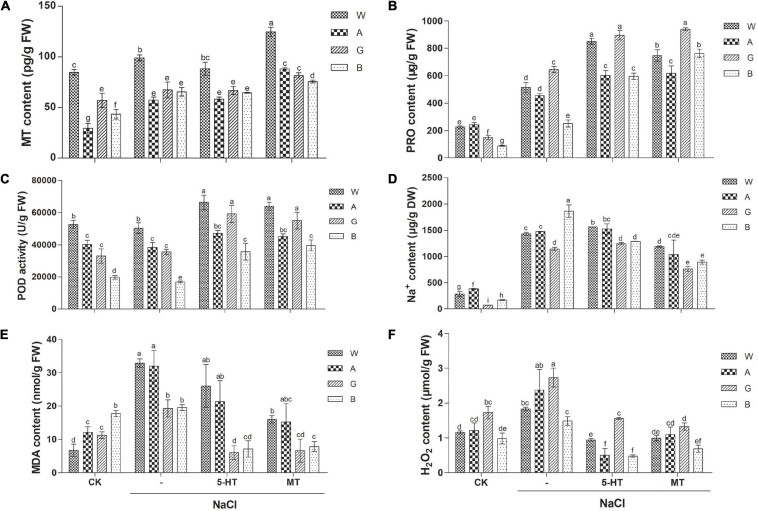
Effects of MT and 5-HT treatment on MT content **(A)**, proline (PRO) content **(B)**, Peroxidase (POD activity) **(C)**, Na^+^ content **(D)**, MDA level **(E)**, and H_2_O_2_ level **(F)** of different alfalfa varieties under salt stress. *n* = 30–60. CK: Water. NaCl: 250 mM. MT: 150 μM. 5-HT: 1 μM. Seeds were germinated for 7 days under different treatments. About 0.01 g of oven-dried seedling was used for the measurement of Na^+^ content and 0.1 g of fresh seedling was used for other measurements in each replicate. Error bars show the SE between biological replicates and a *t*-test was performed between different treatment groups. Different numbers indicated significant differences (*p* ≦ 0.05) between samples. A, Algonquin; B, Bingchi; G, Golden Empress; W, Weiner.

Endogenous MT content was higher in the salt-tolerant variety, Weiner (85 pg/g) than those in the other three varieties (29–57 pg/g), indicating that salt tolerance of Weiner might partly be due to its highest MT level. NaCl treatment slightly increased MT level, while application of MT and 5-HT significantly elevated endogenous MT level in all the four varieties (Figure 3A).

### Both Proline Content and Antioxidant Enzyme Activities Are Positively Correlated With Salt Tolerance Degree of Alfalfa Varieties and Ameliorative Effects of Melatonin

Proline (PRO) is the main osmoregulatory molecule in plants, and the salt tolerance degree of four alfalfa cultivars was positively correlated with their basal PRO content (88–243 μg/g), which was the lowest in the salt-sensitive variety, Bingchi. Salt stress significantly increased PRO content in all four varieties by 1.9–4.3-folds. Both MT and 5-HT treatments significantly increased the PRO content by 1.4–3.0 and 1.3–2.4 folds, respectively (Figure 3B). We speculated that MT and 5-HT conferred salt tolerance by elevating the PRO content in all the four alfalfa varieties, irrespective of their differences in salt tolerance degree.

Peroxidase is one of the antioxidant enzymes with ROS scavenging activity. The basal POD activity is fairly correlated with the salt tolerance level of four alfalfa varieties, namely Wiener > Algonquin > Golden Empress > Bingchi, indicating that the salt tolerance of these alfalfa varieties might be due to their basal POD activity, at least partly. Salt stress did not significantly alter POD activity in all four varieties. Both MT and 5-HT treatment significantly elevated the POD activity by 13–135 and 17–111% compared with the NaCl treatment alone (Figure 3C). We then analyzed the activities of two more antioxidant enzymes, SOD and CAT. Salt stress slightly increased SOD and CAT activities, while both MT and 5-HT treatment further elevated the enzyme activities compared with the NaCl treatment alone (Supplementary Figures 6A,B).

### Melatonin Mitigates Salt-Induced Increases in Na^+^ and MDA Contents

Cellular accumulation of Na^+^ upon salt stress results in ion toxicity and membrane injury. Basal Na^+^ content varies from 72 to 380 μg/g in four alfalfa varieties, and relatively tolerant Algonquin and Weiner have a higher basal level of Na^+^ than the relatively susceptible Bingchi and Golden Empress. Under NaCl treatment, Na^+^ content increased differently in four varieties by 3.9–16-folds, respectively. Under salt stress, Bingchi accumulates the largest concentration of Na^+^; it is spectacular in consideration of its low level of basal Na^+^ content, demonstrating that low Na^+^ efflux activity in Bingchi might be the reason for its highest salt sensitivity. Exogenous application of MT significantly decreased Na^+^ content in all four varieties by 17–52%, demonstrating that MT can alleviate the ion toxicity caused by NaCl stress by accelerating the discharge of Na^+^ in alfalfa seedlings. Compared with MT, 5-HT treatment decreased Na^+^ content only in Bingchi by 31%, while it did not have such effects in the other three varieties, demonstrating that 5-HT alleviated salt stress mainly by the mechanisms other than Na^+^ efflux (Figure 3D). Consistent with Na^+^ change, the K^+^/Na^+^ ratio was decreased after NaCl treatment, while it was increased upon the treatments with MT and 5-HT (Supplementary Figure 6C).

Under adversity, ROS accumulation accelerates the peroxidation of membrane lipid to produce toxic substances, such as an MDA, which is an indicator of lipid damage. Interestingly, basal MDA content was the highest in salt-sensitive Bingchi even if its Na^+^ content was relatively low, while salt-tolerant Weiner had the lowest MDA content although it accumulated a higher Na^+^ level. This might be one of the major contributing factors for the higher salt tolerance of Weiner and the salt sensitivity of Bingchi. NaCl stress significantly increased MDA content compared with the control group. Compared with NaCl treatment, MT treatment reduced MDA content in all the four varieties by 51–66%, while 5-HT decreased MDA content only in relatively sensitive Golden Empress and Bingchi by 69 and 63%, respectively. The results showed that both MT and 5-HT could function as membrane stabilizers by reducing MDA content (Figure 3E).

### Effect of MT as ROS Scavenger on Endogenous H_2_O_2_

Damage of ROS is the common feature of salt stress, and H_2_O_2_ is the main component of ROS, so we investigated the effects of MT on H_2_O_2_ accumulation in alfalfa seedlings. First, the effects of H_2_O_2_ on alfalfa seed germination and seedling growth were determined. H_2_O_2_ exacerbated inhibitory effects of salt stress on alfalfa seed germination, indicating that H_2_O_2_-triggered oxidative stress could negatively affect alfalfa seed germination (Supplementary Figure 7A). In general, H_2_O_2_ treatment had no further aggravating effects on alfalfa seedling growth under salt stress, indicating that oxidative stress represented by H_2_O_2_ mainly influenced seed germination while it did not affect seedling growth (Supplementary Figures 7B–D). However, H_2_O_2_ further reduced the dry weight and relative water content of salt-sensitive variety, Bingchi compared with NaCl alone treatment, indicating that H_2_O_2_ effects are cultivar-dependent and extreme sensitivity of Bingchi is due to many factors including H_2_O_2_ Supplementary Figures 7B,D). Basal H_2_O_2_ contents are comparable between four varieties and NaCl treatment significantly elevated the H_2_O_2_ level in all the four varieties by 49–58%. Both MT and 5-HT treatments significantly decreased H_2_O_2_ levels by 46–54 and 49–79% (Figure 3F). DAB staining more directly shows the accumulation of H_2_O_2_ in seedlings under stress, and the results are consistent with the above data (Supplementary Figure 8).

### Melatonin Promotes Alfalfa Growth Under NaCl Stress in Soil Culture

**FIGURE 4:**
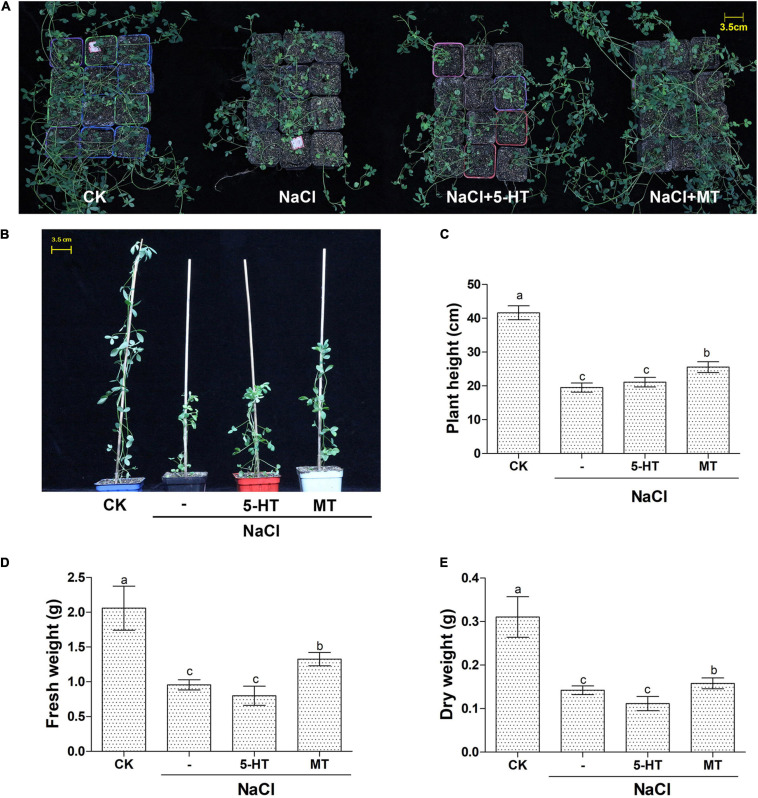
Overall features of soil-grown alfalfa seedlings upon MT and 5-HT treatment with salt stress. Seedlings of alfalfa variety, Bingchi grown in soil pot, were irrigated with 137 mM NaCl for 14 days and were then treated with 150 μM MT or 1 μM of 5-HT together with NaCl, respectively. On the 30th day post-treatments, the pot-grown seedling **(A)** and representative individuals **(B)** from each group were photographed. Scale bar was shown. At least 20 individuals were measured for plant height **(C)**, fresh weight **(D)**, and dry weight **(E)**. Error bars show the SE between biological replicates and a *t*-test was performed between different treatment groups. Different numbers indicated significant differences (*p* ≦ 0.05) between samples.

**FIGURE 5:**
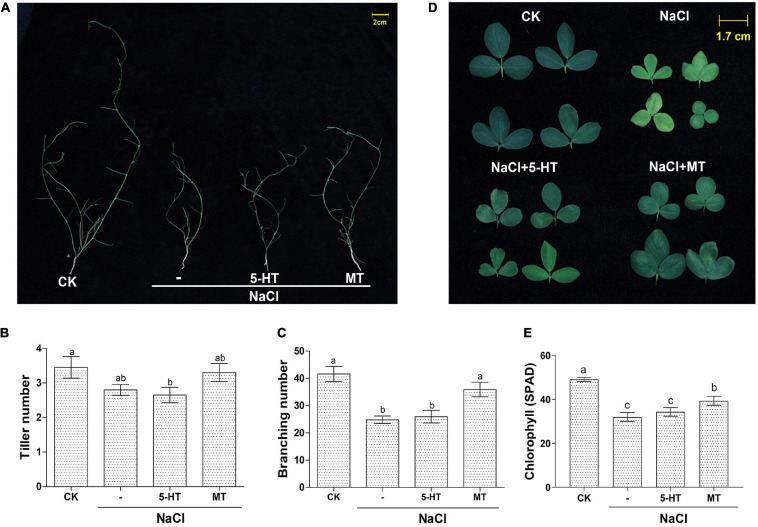
Detailed parameters of soil-grown alfalfa seedlings upon MT and 5-HT treatment with salt stress. Seedlings of alfalfa variety, Bingchi grown in soil pot, were irrigated with 137 mM NaCl for 14 days and were then treated with 150 μM MT or 1 μM of 5-HT together with NaCl, respectively. On the 30th day post-treatments, growth-related parameters were measured. **(A)** Pictures of defoliated plants highlighting the branches; **(B)** Tiller number, *n* = 20; **(C)** Branching number, *n* = 20; **(D)** Representative leaf phenotype; **(E)** Chlorophyll content of the leaf, *n* = 45. Error bars show the SE between biological replicates and a *t*-test was performed between different treatment groups. Different numbers indicated significant differences (*p* ≦ 0.05) between different treatments. The leaves were randomly selected from each treatment, and the relative content of chlorophyll in leaves was detected by a hand-held chlorophyll analyzer (REnQ-IV).

Based on the above experiments using water-cultured seedlings in the early vegetative stage; soil-grown Bingchi was exposed to different treatments to investigate the long-term effects of MT on salt-stressed alfalfa. Bingchi was chosen hereafter as it was the most sensitive variety; therefore, we reasoned that it was the optimal variety for study on ameliorative effects of MT compared with the other three varieties. The growth-related parameters were measured at the mid-bud stage upon different treatments. Overall, NaCl treatment hindered plant growth, MT ameliorated salt stress and improved plant growth, while 5-HT had no effects (Figure 4A). Under salt treatment, the plant height, fresh weight, and dry weight were significantly reduced by 53, 54, and 54%, respectively, compared with the control water treatment group. While 5-HT had no significant effects, MT treatment could significantly improve the tolerance of alfalfa plant to NaCl stress by increasing the plant height, fresh weight, and dry weight by 31, 39, and 11%, respectively, compared with the salt treatment group (Figures 4B–E). Morphologically, salt stress did not reduce tiller number, while it significantly decreased branching number by 40%, while MT treatment recovered the branching number to almost the normal level (Figures 5A–C). Under NaCl stress, the obvious phenomenon of leaf yellowing and withering was observed, and the relative chlorophyll content decreased by 34% compared with the control group. The MT treatment reduced the phenomenon of leaf yellowing and wilting and increased chlorophyll content by 21% compared with NaCl treatment, while 5-HT treatment slightly alleviated the symptom but did not increase the chlorophyll content (Figures 5D,E).

### Putative Melatonin Receptor *MsPMTR1* Is Indispensable for Melatonin Alleviation on Salt Stress in Alfalfa

**FIGURE 6:**
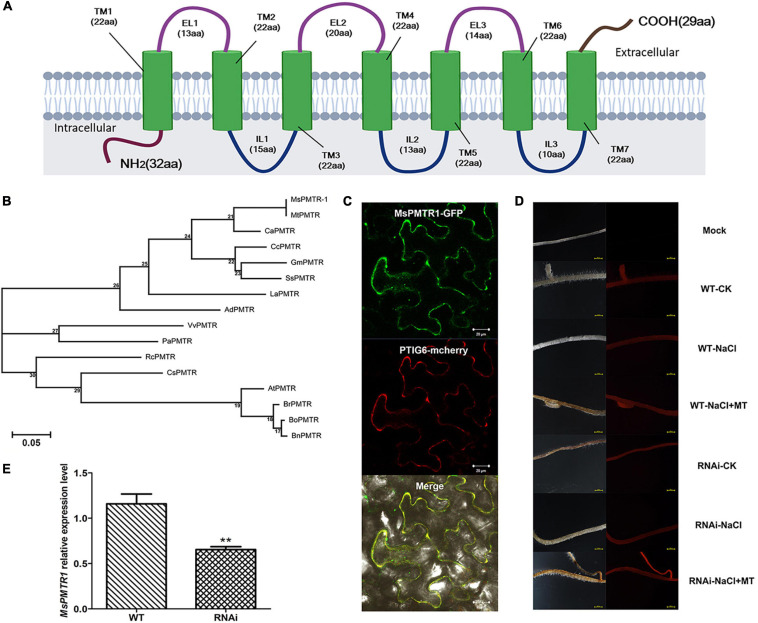
Structural analysis of *MsPMTR1* and silencing of it *via* hairy root transformation. **(A)** Predicted domain architecture of *MsPMTR1* protein. **(B)** Phylogenetic analysis of *PMRT1* homologs from different plant species. *MsPMTR1* sequences were retrieved from *Medicago sativa* transcriptome data. All other sequences were identified from a public database, such as NCBI. The protein sequences were multiple compared using clustalW. MEGA 7.0 is used to build the phylogenetic tree through the maximum likelihood method, and the bootstrap method is used to verify the quality of tree building, with 1,000 times of inspection. *MsPMTR1*, *M*. *sativa*, Cluster-15808.11987; *MtPMTR*, *M*. *truncatula*, P_013469022.1; *CaPMTR*, *Cicer arietinum*, XP_004495767.1; *GmPMTR*, *Glycine max*, XP_006589158.1; *SsPMTR*, *Spatholobus suberectus*, TKY52167.1; *CcPMTR*, *Cajanus cajan*, XP_020209653.1; *LaPMTR*, *Lupinus albus*, KAE9618996.1; *AdPMTR*, *Arachis duranensis*, XP_015939963.1; *VvPMTR*, *Vitis vinifera*, XP_002269068.1; *PaPMTR*, *Prunus avium*, XP_021817586.1; *RcPMTR*, *Ricinus communis*, XP_002512286.1; *CsPMTR*, *Citrus sinensis*, KDO42706.1; *AtPMTR1*, *Arabidopsis thaliana*, OAP02387.1; *BrPMTR*, *Brassica rapa*, XP_009134806.1; *BoPMTR*, *Brassica oleracea*, XP_013629908.1; and *BnPMTR*, *Brassica napus*, XP_013684428.1. **(C)** Subcellular localization of *MsPMTR1*. *Nicotiana benthamiana* leaves were infiltrated with *Agrobacterium tumefaciens* GV3101 containing pEarleyGate103-SL-*MsPMTR1* vector, and the subcellular localization of GFP-fused *MsPMTR1* was observed by confocal microscopy (Zeiss LSM710) under 635 nm red excitation light at 36 hpi. Plasma membrane marker protein, PTIG6 fused with mCherry is shown. **(D)** Confirmation of hairy root transformation by red fluorescent protein (RFP) expression. The left panel is the alfalfa hair root taken under natural light, and the right panel is the alfalfa hair root photographed under green excitation light (Wavelength = 558 nm). **(E)** Detection of *MsPMTR1* gene knockdown by qRT-PCR experiment. The housekeeping gene, *MsActin* was used as the reference. Data represent mean values for three independent biological replicates. Standard errors are indicated by vertical bars. Asterisks indicate a statistical difference of the value at the **P < 0.01 levels as determined by a *t*-test.

The above experiments indicated that MT could alleviate salt stress on alfalfa seed germination and plant growth. In *A*. *thaliana*, a putative MT receptor named, CAND2/PMTR1 was reported to mediate MT signaling (Wei et al., 2018). By homology searching, we identified a highly homologous gene of *CAND2/PMTR1* from *M*. *sativa* and we named it *MsPMTR1*. The *MsPMTR1* is a predicted membrane-localized protein with seven transmembrane domains (Figure 6A). The *MsPMTR1* showed 71% sequence identity to *CAND2/PMTR1* (Figure 6B). Subcellular localization analysis by green fluorescent protein (GFP)-fused expression of *MsPMTR1* in *Nicotiana Benthamiana* transient expression system showed that *MsPMTR1* localized at the cell membrane, demonstrating its possible role as a membrane-localized receptor (Figure 6C).

**FIGURE 7:**
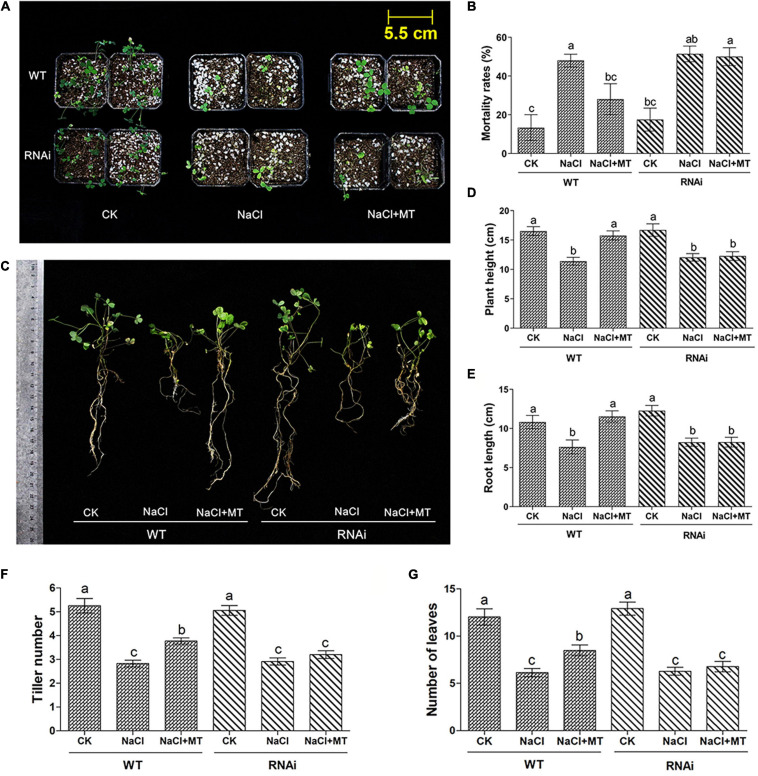
The phenotype of alfalfa seedlings under salt stress and MT treatment upon *MsPMTR1* gene knockdown. **(A)** The phenotype of a representative individual from each treatment. **(B)** Mortality rates, *n* = 24. **(C)** The Phenotype of representative seedling from each treatment. **(D)** Plant height, *n* = 30. **(E)** Root length, *n* = 30. **(F)** Tiller number per seedling, *n* = 35. **(G)** The Number of leaves per seedling, *n* = 35. Error bars show the SE between biological replicates and a *t*-test was performed between different treatment groups. Different numbers indicate significant differences (*p* ≦ 0.05) between different treatments. WT, control pKGWRR-RFP transformed seedling. RNAi, *MsPMTR1*-silenced seedling. CK, control water treatment. NaCl, 137 mM NaCl. NaCl + MT, 137 mM NaCl + 150 μM MT.

We investigated the involvement of *MsPMTR1* in MT-mediated salt tolerance to further ascertain the ameliorative role of MT on salt-stressed alfalfa growth. We generated *MsPMTR1*-silenced alfalfa variety, Bingchi *via* hairy root transformation, and the transgenes were verified by RFP expression as pKGWRR vector carried *RFP* gene (Figure 6D). The RT-qPCR analysis showed that the mRNA level of *MsPMTR1* was significantly reduced by 44% in the *MsPMTR1*-silenced plants compared with the control group (Figure 6E). The transgenic lines were transferred to soil pot and were treated by 137 mM NaCl with or without MT to investigate its relieving effects. The seedlings almost perished under salt stress, and MT treatment restored plant growth in control lines but it did not show ameliorative effects in *MsPMTR1*-silenced plants (Figure 7A). In the control lines, NaCl treatment caused 35% higher seedling mortality than the control group and reduced the plant height and root length by 35 and 27%, respectively, while MT treatment fully recovered plant growth to the control level with regards to the above parameters. Compared with this, the MT treatment could not recover salt-induced seedling mortality and growth retardation in *MsPMTR1*-silenced alfalfa seedlings (Figures 7B–E). In the control lines, salt stress caused a 46 and 49% reduction in tiller number and leaf number, respectively, compared with mock treatment, while MT treatment partially recovered them by 33 and 38% compared with the NaCl-treated sample. On the contrary, MT treatment could not recover salt-induced decrease in tiller number and leaf number in *MsPMTR1*-silenced alfalfa seedlings (Figures 7F,G).

**FIGURE 8:**
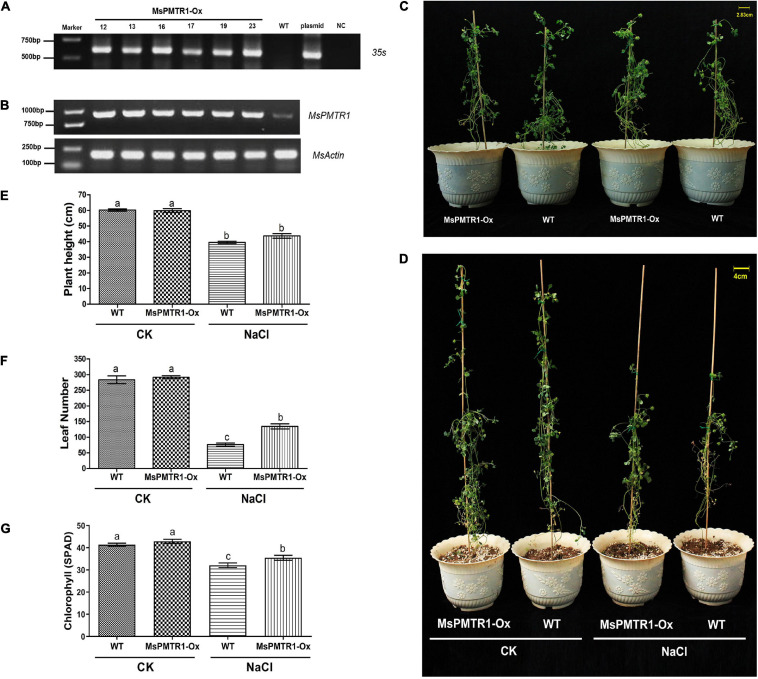
Hairy root overexpression of *MsPMTR1* in *M*. *sativa* and phenotypic analysis of transgenic lines under salt stress. **(A)** PCR detection of transgenic alfalfa lines. *MsPMTR1*-Ox, *MsPMTR1* overexpressed plants, and different lines are indicated. WT, wild-type plant. Plasmid, pCambia1307-*MsPMTR1* plasmid as a positive control for PCR. NC, negative control for PCR. The sizes of DM2000 markers are shown on the left. **(B)** RT-PCR confirmation of *MsPMTR1* expression. *MsActin* expression is detected as endogenous control. **(C)** The phenotype of a representative individual from transgenic and wild-type plants before treatment. **(D)** The phenotype of a representative individual from transgenic and wild-type plants after 21 days of treatment. CK, control water treatment. NaCl, 137 mM NaCl treatment. **(E–G)** plant height, leaf number, and chlorophyll content after 21 days of control and NaCl treatment on both transgenic and wild-type plants. Three biological replicates were performed for each assay. Error bars show the SE between biological replicates and a *t*-test was performed between different treatment groups. Different numbers indicate significant differences (*p* ≦ 0.05) between different treatments.

**FIGURE 9:**
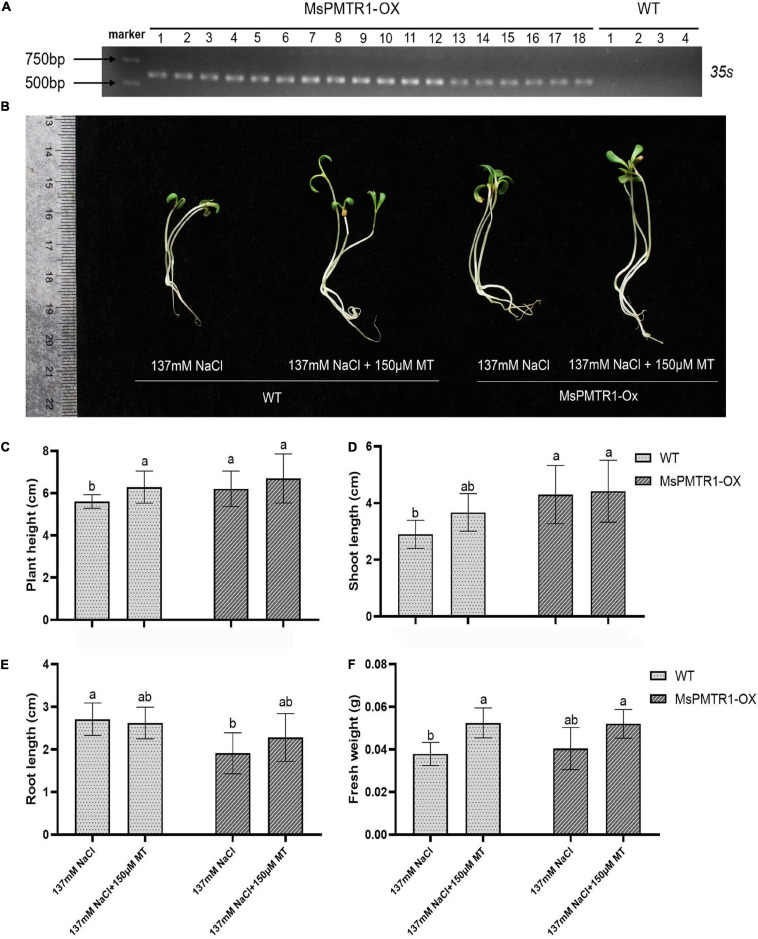
Stable overexpression of *MsPMTR1* in *M*. *truncutula* and phenotypic analysis of transgenic lines under salt stress. **(A)** PCR detection of transgenic alfalfa lines. Different lines are indicated. WT, wild-type alfalfa cultivar R108. The sizes of DM2000 markers are shown on the phenotype of a representative individual from transgenic and wild-type plants under NaCl and MT treatment. **(C–F)** plant height, shoot length, root length, and fresh weight. Three biological replicates were performed for each assay. Error bars show the SE between biological replicates and a *t*-test was performed between different treatment groups. Different numbers indicate significant differences (*p* ≦ 0.05) between different treatments.

We generated *MsPMTR1* overexpression alfalfa *via* hairy root transformation, and the transgenes were verified by genomic DNA PCR by 35S promoter-specific primers (Figure 8A). The RT-PCR analysis showed that the mRNA level of *MsPMTR1* was highly elevated in *MsPMTR1*-overexpressed plants (Figure 8B). The seedlings were grown in a soil pot and were treated with 137 mM NaCl for 21 days. The transgenic lines displayed no obvious phenotypic changes under normal conditions (Figure 8C), while they showed better growths when stressed with NaCl compared with wild-type plants (Figure 8D). Under NaCl treatment, *MsPMTR1*-overexpressed plants showed increased leaf number and chlorophyll content compared with wild-type plants although they did not differ in plant height (Figures 8E–G). Furthermore, *MsPMTR1*-overexpression stable lines were generated for *M*. *truncutula*, as a more robust genetic transformation system was available in *M*. *truncutula* than in *M*. *sativa*. The transgenes were confirmed by genomic DNA PCR by 35S promoter-specific primers (Figure 9A). Overall, the transgenic lines showed better growth performances under salinity compared with wild-type plants, with regards to plant height, shoot length, and fresh weight (Figures 9B–F). Consistent with the results in *M*. *sativa*, MT treatment ameliorated salt stress on *M*. *truncutula*, although to a lesser extent (Figures 9B–F). Furthermore, another independent transgenic line showed the same phenotypes (Supplementary Figure 9). Taken together, functional analyses show that *MsPMTR1* might be a possible MT receptor in *M*. *sativa*, and MT action is mediated through membrane-localized *MsPMTR1* perception, especially with regard to its salt-ameliorative effects.

### Molecular Mechanisms of Melatonin Alleviation on Salt Stress in Alfalfa

**FIGURE 10:**
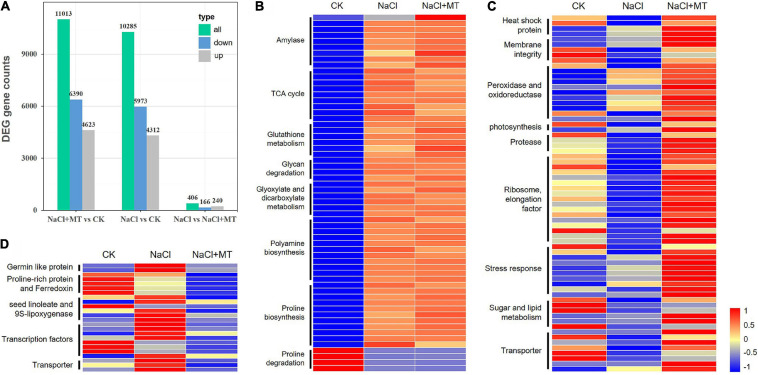
Transcriptome responses of alfalfa seedling after treatment with NaCl and MT. CK, Water. NaCl, 250 mM. NaCl + MT, 250 mM NaCl + 150 μM MT. Seeds were germinated for 10 days under different treatments, and total RNAs of root tissue with three biological replicates from each treatment were used for transcriptome sequencing and identification of differentially expressed genes (DEGs). **(A)** The number of DEGs in different comparisons. “Up” represents upregulated genes and “Down” represents downregulated genes. **(B)** The DEGs showed similar expression patterns in NaCl and NaCl + MT groups compared with control (CK). **(C)** The upregulated genes in NaCl + MT compared with NaCl alone. **(D)** The downregulated genes in NaCl + MT compared with NaCl alone. Hierarchical cluster heatmap was drawn by MeV software, and log2 (RPKM) values for the DEGs are indicated in different colors. Gene annotation is shown on the left.

To investigate the molecular mechanisms underlying alfalfa responses to salt stress and ameliorative effects of MT, transcriptome sequencing (RNA-seq) was performed to identify DEGs under NaCl and MT treatments. The salt-sensitive alfalfa variety, Bingchi was treated with control water (Kang et al., 2010), NaCl, and NaCl + MT, respectively. Around 10,000s DEGs were obtained by NaCl vs. control (CK) and NaCl + MT vs. CK analyses, and most (8,445) of these DEGs showed similar patterns in NaCl and NaCl + MT groups compared with CK, indicating the regulatory predominance of NaCl treatment on gene expression (Figure 10A). The genes involved in energy metabolism, such as “starch degradation (amylase),” “glycan degradation,” “glyoxylate and dicarboxylate metabolism,” and tricarboxylic acid (TCA) cycle are significantly upregulated in NaCl and NaCl + MT groups compared with CK. Stress-related pathway genes, such as PRO biosynthesis gene, pyrroline-5-carboxylate synthetase (*P5CS*), polyamine biosynthesis genes, glutathione S-transferase (*GST*), and *GR* genes were highly induced, while PRO degradation gene, proline dehydrogenase (*ProDH*) were downregulated in NaCl and NaCl + MT groups compared with CK (Figure 10B).

Then, we focused on DEGs between NaCl + MT and NaCl groups to investigate the regulatory role of MT on gene expression. About 406 DEGs were obtained by comparing NaCl + MT with NaCl, of which 166 genes were upregulated and 240 genes were downregulated (Figure 10A). Several basal metabolic pathways, such as “ribosome, elongation factor,” “sugar and lipid metabolism,” and “photosynthesis” genes are inhibited by salt treatment and reversed to normal level by simultaneous MT treatment in the NaCl + MT sample. The genes encoding stress-related proteins, such as heat shock protein, peroxidase/oxidoreductase, and protease are suppressed by NaCl and reversed by MT treatment. Salt stress generally disrupts membrane structure, and several “membrane integrity” related genes were downregulated by NaCl treatment, while MT recovered their expression in the NaCl + MT sample. Several genes encoding transporters of water (aquaporin) and small molecule metabolites, such as amino acid and sugar, ions, such as chloride, ferric iron, and proton, were inhibited by NaCl stress while they were recovered by MT treatment. Several abiotic stress response-related genes, such as dehydrin, a dehydration responsive element (*DRE*), auxin response factor (*ARF*), *HD-ZF*, *MYB*, and remorin (*REM*), were repressed by NaCl treatment while they were induced by MT treatment (Figure 10C). Compared with the above-mentioned genes which were upregulated in NaCl + MT, a variety of genes were downregulated in NaCl + MT compared with the NaCl group. Several ethylene response factor (*ERF*) genes and *NPR1*, a well-known SA receptor gene, are repressed by MT treatment. Seed linoleate and 9S-lipoxygenase could elevate the level of polyunsaturated fatty acid (PUFA) to promote ROS accumulation (Bell and Mullet, 1991). Expression of several linoleates and 9S-lipoxygenase genes, such as *LOX1_5* and *LOX2S*, were elevated by salt treatment, while they were downregulated by MT treatment. Contrary to the increase in PRO synthesis gene expression after NaCl and MT treatment, several proline-rich protein (*PRP*) gene expressions were downregulated by MT treatment. Some PRP proteins seem to directly interact with ferredoxin and are reported to play negative roles in plant abiotic stress responses in tomatoes (Li et al., 2016). Consistent with this, several ferredoxin encoding genes are suppressed by NaCl treatment and further inhibited by MT treatment. Several genes encoding the transporters for calcium, sodium, and lipid were downregulated by MT treatment. Some germin-like proteins tended to increase ROS accumulation in plants (Beracochea et al., 2015), and these genes were highly induced by NaCl, while they were inhibited by MT treatment (Figure 10D). More details of the above-listed DEGs are provided in Supplementary Table 2. We selected 20 unigenes to perform expression analysis to confirm the reliability of transcriptome data. The RT-qPCR experiments indicated that the expression pattern of 17 out of 20 selected unigenes was consistent with the transcriptome data (Supplementary Figure 10).

## Discussion

Improvement of salt tolerance in crop plants is essential for sustainable agriculture. Elucidation of the salt tolerance mechanism of plants appears to be fundamental to this aim. Identification of tolerance-promoting chemical agents, which are either of plant origin or chemically designed, would provide a better option for improving the salt tolerance of the crops in the experimental condition. Although identified first in animals, MT was found to be present in broad spectra of plant species and even in unicellular organisms (Hardeland and Poeggeler, 2003; Zhang et al., 2015). In legume species, such as *Vicia faba* and soybean, seed priming with MT was reported to promote plant growth and enhance salt tolerance (Dawood and Gergis, 2014; Wei et al., 2015). Intriguingly, an unusually higher abundance of melatonin, 2 ng/g–1.1 μg/g, has been reported in plants than in animals (Nawaz et al., 2016). Melatonin content was reported to be as high as 16 ng/g in alfalfa seeds, and the content dramatically decreased to 133 pg/g after germination, showing its role during germination (Aguilera et al., 2015). We identified high-tolerant (Weiner), medium-tolerant (Algonquin and Golden Empress), and sensitive (Bingchi) varieties to pursue our study. Noteworthy, Algonquin was considered to be a salt-sensitive cultivar in some literature (Xiong et al., 2018), while it appeared as a medium-tolerant variety in our experimental condition. Bingchi showed more sensitivity compared with Algonquin, suggesting that Bingchi we used in this study is a very sensitive alfalfa cultivar. The MT content is the lowest in Bingchi among four alfalfa varieties, so it might contribute to its highest salt sensitivity. Supply of exogenous MT and its precursor 5-HT fully recovered the germination of NaCl-stressed alfalfa seedlings to the control level regardless of the salt tolerance ability of four alfalfa varieties, demonstrating that the ameliorative role of MT on salt-stressed seed germination is a general and cultivar-independent effect in alfalfa. The promotive effects of MT on seed germination were also reported in several plant species, such as cucumber, maize, cotton, and *A*. *thaliana* after salt or cold stress (Zhang et al., 2014b; Hernández et al., 2015; Cao et al., 2019; Chen et al., 2020). The5-HT was also reported to alleviate salt stress in rice (Gupta and De, 2017). Both MT and 5-HT could prevent water loss by increasing the relative water content and fresh weight of NaCl-stressed alfalfa seedlings, demonstrating that they both might function to inhibit the osmotic stress caused by salt stress. The 5-HT remarkably recovered both root and shoot growth while MT had an only moderate effect in the early vegetative stage, suggesting that they might function *via* different mechanisms with regards to alleviating salt-stressed alfalfa growth, as is different from their shared effect on alfalfa seed germination. On the contrary, MT showed higher growth-stimulating effects than 5-HT in soil culture experiment in mid bud stage. Indeed, 5-HT itself seems to have divergent functions from MT although it was the well-known precursor of MT (Erland et al., 2015, 2018). The growth-promoting action of MT was reported in *A*. *thaliana*, wheat, and rice under cold stress, and the effects were manifested by increasing fresh weight, root length, plant height *via* ROS scavenging and preservation of chlorophyll content (Kang et al., 2010; Bajwa et al., 2014; Turk et al., 2014).

Proline is an osmoregulatory substance in plants, therefore, PRO content is a key physiological indicator of plant tolerance to salt stress (Mansour and Ali, 2017). Basal PRO contents of four alfalfa varieties are closely correlated with their salt tolerance ability, demonstrating that it might contribute to their degree of salt tolerance. NaCl treatment significantly elevates PRO content, showing that PRO content increase is an adaptive response of alfalfa seedling to salt stress. Increased mRNA level of PRO synthesis key gene *P5CS* and decrease in proline degradation gene *ProDH* expression after salt stress and MT treatment mechanistically explains the increase in PRO content. Consistently, MT treatment significantly reduced endogenous Na^+^ accumulation and sodium transporter gene expression, supporting its regulatory mechanism *via* ionic stress reduction. Salt stress causes ROS accumulation, and the plant responds to it by the activation of ROS scavenging enzymes, such as POD (Adriano et al., 2015). Similar to basal PRO content, basal POD activities are positively related to the salt-tolerance ability of four alfalfa varieties, demonstrating that basal POD activity might be another reason for their degree of tolerance to salt stress. Although NaCl did not alter it, both MT and 5-HT treatments significantly elevated POD activity, indicating that increased POD activity is another salt-tolerance mechanism mediated by MT and 5-HT. Consistent with increasing POD activity, transcriptome analysis showed that expression of several genes encoding peroxidase and oxidoreductase were consistently upregulated by MT treatment compared with NaCl treatment, demonstrating that MT could transcriptionally regulate these genes to elevate POD activity. In line with the increase in POD activity, MT and 5-HT treatment significantly reduced the accumulation of endogenous H_2_O_2_ and MDA, both of which are indicators of ROS damage. In the soybean culture system, exogenous application of PRO improved salt tolerance by enhancing both POD and SOD activities (Yan et al., 2000; Bai and Wang, 2002), indicating that PRO and POD have interacting networks.

Plant response to salt stress is an energy-consuming process. RNA-seq analysis showed that the expression of unigenes involved in basal metabolism of sugar degradation and energy production was upregulated by salt stress and MT treatment. Glycan degradation and starch catabolism are involved in the production of free sugar. One should note that germinating seeds acquire their energy by metabolizing triacylglycerol (TAG) and starch stored in the seeds. In our transcriptome analysis, “glyoxylate and dicarboxylate metabolism” pathway genes are enriched after NaCl and MT treatment and this pathway is responsible for converting lipid energy to synthesize carbohydrates to sustain seedling growth. These results are similar to the data produced by Zhang et al. (2017), where salt-stressed cucumber seedlings accumulated glyoxylate cycle-related enzymes under MT treatment. Amylases are responsible for starch metabolism, and we found that NaCl and MT treatment-induced amylase gene expression. Increased amylase activities were also observed during seed germination of salt-stressed cucumber after MT treatment (Zhang et al., 2017). As all the above pathways in plants are responsible for the elevation of free sugars and elevated free sugars are reported to enhance salt tolerance in plants, these might represent the resistance responses of alfalfa plants against salt stress. Our data are consistent with the results in turfgrass, *Cynodon dactylon*, in which MT treatment increases the tolerance to salt, drought, and cold stress by a higher accumulation of amino acids, organic acids, and free sugars (Shi et al., 2015). The TCA cycle is a basic metabolic pathway responsible for the production of cellular energy by utilizing monosaccharides *via* aerobic oxidation, and the expression of genes related to the TCA cycle was upregulated by both NaCl and MT treatment. Two other cellular molecules, such as GSH and polyamine, are involved in plant stress responses (Noctor and Foyer, 1998). Expression of genes encoding synthesis of polyamine was reported to participate in salt inhibition in plant species, such as wheat and cucumber (Ke et al., 2018; Seo et al., 2019; Yin et al., 2019).

Pathways related to “ribosome synthesis” and “photosynthesis related” genes are inhibited by salt treatment and reversed to normal level by the simultaneous MT treatment in the NaCl + MT sample. These demonstrate that NaCl stress retards seedling growth by inhibiting protein synthesis and photosynthesis, while MT treatment alleviates salt stress by elevating these cellular processes. This is consistent with the chlorophyll content increase and in the alleviation of salt-induced leaf yellowing by MT treatment in soil culture experiment in this study. Heat shock proteins (HSPs), especially HSP70, enhance plant abiotic stress tolerance (Jiang et al., 2020), and MT treatment increased the expression of several *HSP* genes compared with both CK and NaCl samples. Salt stress generally disrupts membrane structure, and several “membrane integrity” related genes are downregulated by NaCl treatment, while MT recovered their expression in the NaCl + MT sample. Although expression of genes for free sugar production and energy metabolism is commonly upregulated in both NaCl and NaCl + MT samples, several genes encoding glycogen phosphorylase are significantly downregulated by NaCl treatment while they were distinctively recovered by MT treatment. Proteases, such as *A*. *thaliana* aspartic protease *APA1* gene (Sebastián et al., 2020) and papain-like cysteine proteinases (Fischer et al., 2000; Müntz et al., 2001) enhance drought tolerance and seed germination in several plant species, and expressions of these genes were inhibited by salt stress while they were induced by MT treatment. Several abiotic stress-response related genes, such as *DRE* (Liu et al., 2015), *ARE*, *HD-ZF*, *MYB*, and *REM* (Yue et al., 2014), were repressed by NaCl treatment while they were induced by MT treatment. Compared to the above upregulated genes in MT + NaCl, a variety of genes were downregulated in MT + NaCl compared with NaCl alone. The 9S-lipoxygenase oxidizes linoleic acid and linolenic acid elevate the level of nonsaturated fatty acid (Zaman et al., 2018), and the expression of several seed-specific linoleates, such as 9S-lipoxygenase genes, was elevated by salt treatment, while they were downregulated by MT treatment. It might contribute to salt tolerance by MT decreasing MDA content as alpha-linolenic acid is a precursor of MDA (Li et al., 2016). Our RNA-seq data identified some common MT-responsive genes which were reported in other plant species. For example, Liu et al. (2020) showed that K^+^ transporter genes were induced upon MT treatment in rice seedlings, and it was consistent with our data of K^+^/Na^+^ increases upon MT treatment. Zhang et al. (2014c) identified the same MT-responsive TF family genes, such as *MYB*, *WRKY*, *ERF*, and *NAC* from cucumber root as in our present study. Zhan et al. (2021) reported that the same TF genes shown above and other genes involved in photosynthesis, sugar metabolism, and GSH metabolism in MT-treated *Abelmoschus esculentus*. Collectively, the data from the above literatures and our study indicate the widespread and conserved effects of MT in different plant species.

In the pot experiment, the growth and biomass of pot-cultured alfalfa plants were remarkably retarded by salt treatment, while MT treatment could enhance the alfalfa plant salt tolerance by increasing the growth parameters. Salt stress caused remarkable senescence phenomenon of leaf yellowing and withering and decreased chlorophyll content, whereas MT alleviated this symptom by increasing the chlorophyll content. In our transcriptome data, the genes involved in photosynthesis are highly enriched after MT treatment, supporting the photosynthesis activation of MT. Growth-promoting effects of MT were also reported in other plant species. For example, MT promoted coleoptile growth in canary grass, wheat, barley, and oats (Hernández-Ruiz et al., 2005). The MT-accelerated root growth of *Brassica juncea* (Chen et al., 2009). The MT increased corn yield and also improved soybean yield by increasing seed number and pod number, and also increased their salt tolerance (Tan et al., 2012; Wei et al., 2015). All of these above data indicate the salt tolerance promotion of MT in alfalfa in the aspects of seed germination and photosynthesis activation. To sum up, MT ameliorates NaCl-triggered osmotic stress by increasing the PRO content, ion toxicity by Na^+^ efflux, oxidative stress by MDA decreasing and ROS (H_2_O_2_) scavenging. Mechanistically, MT also induces lipid, sugar, and amino acid metabolism genes to provide energy and sustain plant growth.

Melatonin plays a broad role in abiotic stress tolerance, but the cell perception of MT to trigger downstream signaling is unclear. The MT receptors were identified in an animal in 1994 and the first animal melatonin receptor, Mel1c is a G protein-coupled receptor (GPCR) (Ebisawa et al., 1994). In line with the limited functional study of MT in plants, the reports on its receptor are also lagged in plants compared with animal research. AtCAND2/PMTR1, a membrane-localized protein with seven transmembrane helices, which mediates stomatal closure *via* H_2_O_2_ and Ca^2+^ signaling cascade, is referred to be the first phyto-melatonin receptor in *A*. *thaliana* (Wei et al., 2018). To further illustrate the MT signaling pathway in alfalfa, we identified *MsPMTR1*, a highly homologous gene of *CAND2/PMTR1*, from *M*. *sativa* by homology searching. *MsPMTR1* and AtCAND2 showed sequence identity of 71%, which is considerably high as some receptors with lower identity displayed the same function. For example, Zhang et al. (2004) reported a functional ethylene receptor NTHK2 from tobacco and it showed only 46.4% identity with a well-established *Arabidopsis* ethylene receptor, ETR2. The *MsPMTR1* was membrane-localized and silencing of *MsPMTR1* abolishes the alleviating effects of MT on salt stress-induced growth abnormality in alfalfa. Therefore, *MsPMTR1* might be a possible MT receptor in alfalfa and MT action is mediated through *MsPMTR1* perception, especially with regards to its salt inhibitory role. To our knowledge, this is the second report on MT receptors in plants, and we validate the data of Wei et al. (2018) on MT receptor in a plant species besides model organism *A*. *thaliana*). However, a recent report argued the authenticity of AtCAND2 as a melatonin receptor (Lee and Back, 2020). Lee and Bacl provided evidence that AtCAND2 was localized in the cytoplasm rather than the plasma membrane, which was reported by Wei et al. Furthermore, they showed that melatonin-mediated mitogen-activated protein kinase (MAPK) activation and defense gene induction were not abolished in *Arabidopsiscand2* knockout mutant lines (Lee and Back, 2020). However, another study showed that CADN2 was essential for MT-triggered osmotic stress tolerance in *Arabidopsis*, supporting the role of CAND2 as a putative MT receptor (Wang et al., 2021). Our data indicate the membrane localization of *MsPMTR1*. We provide some functional evidence of *MsPMTR1* as a putative melatonin receptor. However, biochemical studies are required for confirmation of *MsPMTR1* as a real receptor. Receptor identification and mechanistic elucidation are important for the tailoring of plant hormones to engineer stress-tolerant crop cultivation. Taken together, we provide compelling evidence for the ameliorative effects of MT on salt-stressed alfalfa seed germination and seedling growth by in-depth physiological, transcriptional, and downstream signaling analyses using both salt-tolerant and sensitive varieties.

## Data Availability Statement

The datasets presented in this study can be found in online repositories. The names of the repository/repositories and accession number(s) can be found below: https://www.ncbi.nlm.nih.gov/, PRJNA742658.

## Author Contributions

HW designed the experiments and wrote the manuscript. YN and LP designed and supervised some of the experiment. RY and TZ performed most of the experiments. PD and RY analyzed the data and prepared the figures. JF, YF, XM, WL, YW, QZ, AL, RW, and FY performed some of the experiments. All authors contributed to the article and approved the submitted version.

## Conflict of Interest

The authors declare that the research was conducted in the absence of any commercial or financial relationships that could be construed as a potential conflict of interest.

## Publisher’s Note

All claims expressed in this article are solely those of the authors and do not necessarily represent those of their affiliated organizations, or those of the publisher, the editors and the reviewers. Any product that may be evaluated in this article, or claim that may be made by its manufacturer, is not guaranteed or endorsed by the publisher.

## Footnotes

1
https://dataview.ncbi.nlm.nih.gov/object/PRJNA742658?reviewer=spik7cgmmaite2hqebas4rruil

## Supplementary Material

Supplementary Figure 1 Phenotypic observation of germination test for Figure 1.

Supplementary Figure 2 Salt-stress reduced growth parameters of different alfalfa varieties. CK: Water. NaCl: 200, 225, 250, 275, and 300 mM. For each replicate, one hundred alfalfa seeds were surface-sterilized, spread evenly on two layers of filter paper which were presoaked with 5 ml treatment solution, and placed in a Petri dish at 20°C in an illuminating incubator. **(A)** Fresh weight. **(B)** Dry weight. **(C)** Relative water content. *n* = 40. A *t*-test was performed between samples in different treatment groups. Different numbers indicate significant differences (*p* ≦ 0.05) between different treatments. A, Algonquin; B, Bingchi; G, Golden Empress; W, Weiner.

Supplementary Figure 3 Different concentrations of MT treatment. The experiment was carried out as the experiment in Figure 1 unless adding different concentrations of MT.

Supplementary Figure 4 Different concentrations of 5-HT treatment. The experiment was carried out as the experiment in Figure 1 unless adding different concentrations of 5-HT.

Supplementary Figure 5 Effects of combined treatment on fresh weight **(A)**, dry weight **(B)**, and relative water content **(C)** of different alfalfa varieties under salt stress. CK: Water. NaCl: 250 mM. MT: 150 μM. 5-HT: 1 μM. Seeds were germinated for 10 days under different treatments, and 30–60 seedlings from each replicate were used for measurement. Water content = [(Fresh weight − Dry weight)/Fresh weight] × 100%. Error bars show the SE between biological replicates and a *t*-test was performed between different treatment groups. Different numbers indicate significant differences (*p* ≦ 0.05) between samples. A, Algonquin; B, Bingchi; G, Golden Empress; W, Weiner.

Supplementary Figure 6 Measurement of SOD **(A)**, CAT **(B)**, and K^+^/Na^+^ **(C)**. The experiment was carried out as the experiment in Figure 3.

Supplementary Figure 7 Effects of H_2_O_2_ treatment on germination and growth of different alfalfa varieties. CK: Water; NaCl: 250 mM. For each replicate, one hundred alfalfa seeds were surface-sterilized, spread evenly on two layers of filter paper which were presoaked with 5 ml treatment solution, and placed in a Petri dish at 20°C in an illuminating incubator. **(A)** Germination rate. Germinated seeds were calculated on the 10th day and the germination rate was determined. Three biological replicates were performed and error bars show the SE values. **(B)** Relative water content. **(C)** Fresh weight. **(D)** Dry weight. *n* = 30. A *t*-test was performed between samples in different treatment groups. Different numbers indicate significant differences (*p* ≦ 0.05) between different treatments. A, Algonquin; B, Bingchi; G, Golden Empress; W: Weiner.

Supplementary Figure 8 DAB-staining of alfalfa seedlings.

Supplementary Figure 9 Phenotypic observation of another *M*. *truncutula* line. The experiment was carried out as the experiment in Figure 9.

Supplementary Figure 10 The qRT-PCR confirmation of selected unigenes from transcriptome data. The mRNA levels of 20 unigenes were detected by qRT-PCR experiment using gene-specific primers. The housekeeping gene, *MsActin* was used as the reference. Data represent mean values for three independent biological replicates. SE is indicated by vertical bars. Different numbers indicate significant differences (*p* ≦ 0.05) between different treatments. CK, Water. NaCl, 250 mM. NaCl + MT, 250 mM NaCl + 150 μM MT.

Supplementary Table 1 The qRT-PCR primers for the detection of unigene expression.

Supplementary Table 2 The statistical output of RNA-seq data for selected differentially expressed genes (DEGs) in Figure 10.
