# Supplementary figures and images for: Melatonin Enhances Seed Germination and Seedling Growth of Medicago sativa Under Salinity via a Putative Melatonin Receptor MsPMTR1
Source: Front Plant Sci. 2021 Aug 17;12:702875. doi: 10.3389/fpls.2021.702875 (PMC8418131; doi:10.3389/fpls.2021.702875)

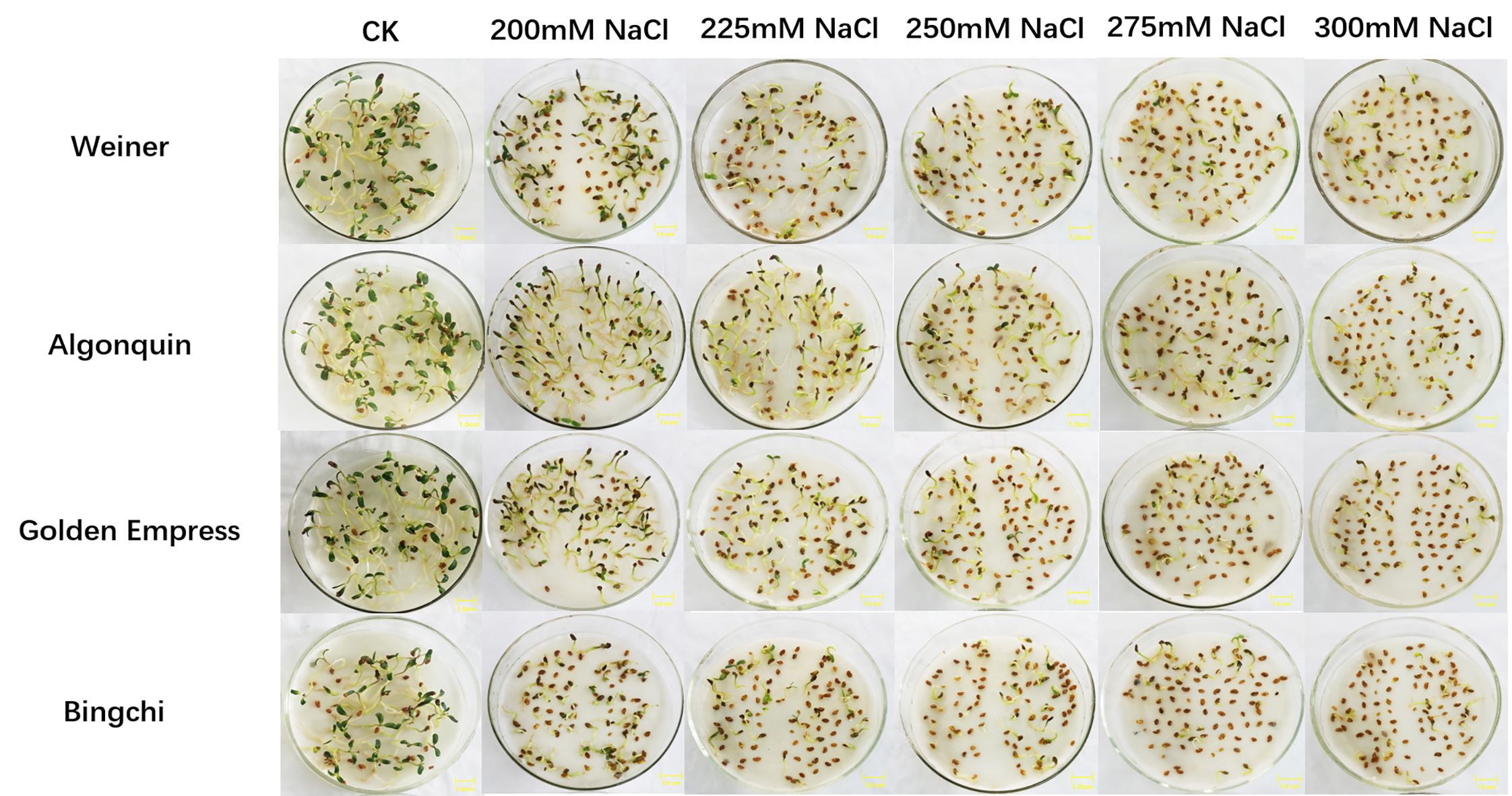

Supplement: Supplementary Figure 1 — Phenotypic observation of germination test for Figure 1. [file Image_1.JPEG]

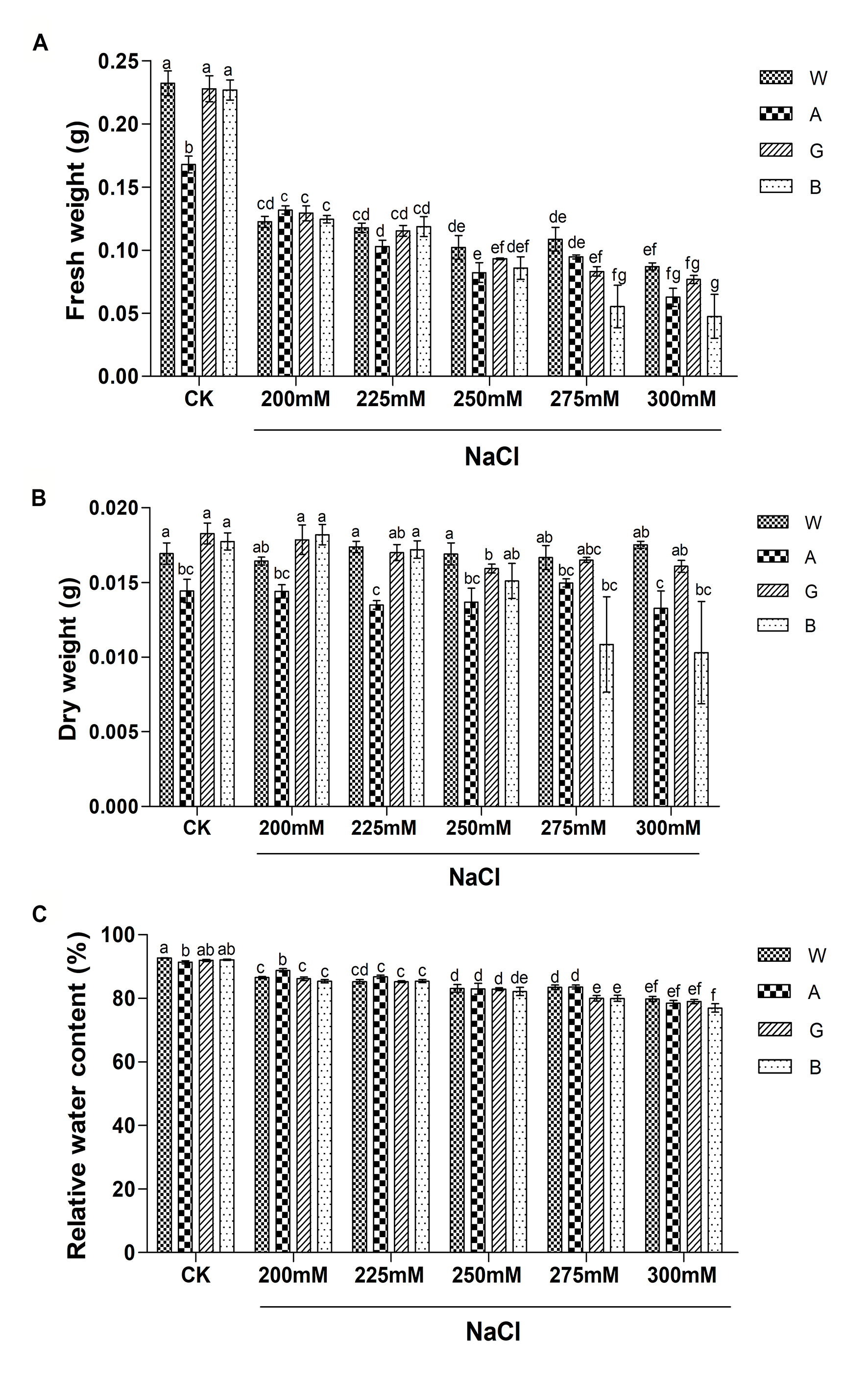

Supplement: Supplementary Figure 2 — Salt-stress reduced growth parameters of different alfalfa varieties. CK: Water. NaCl: 200, 225, 250, 275, and 300 mM. For each replicate, one hundred alfalfa seeds were surface-sterilized, spread evenly on two layers of filter paper which were presoaked with 5 ml treatment solution, and placed in a Petri dish at 20°C in an illuminating incubator. (A) Fresh weight. (B) Dry weight. (C) Relative water content. n = 40. A t-test was performed between samples in different treatment groups. Different numbers indicate significant differences (p ≦ 0.05) between different treatments. A, Algonquin; B, Bingchi; G, Golden Empress; W, Weiner. [file Image_2.JPEG]

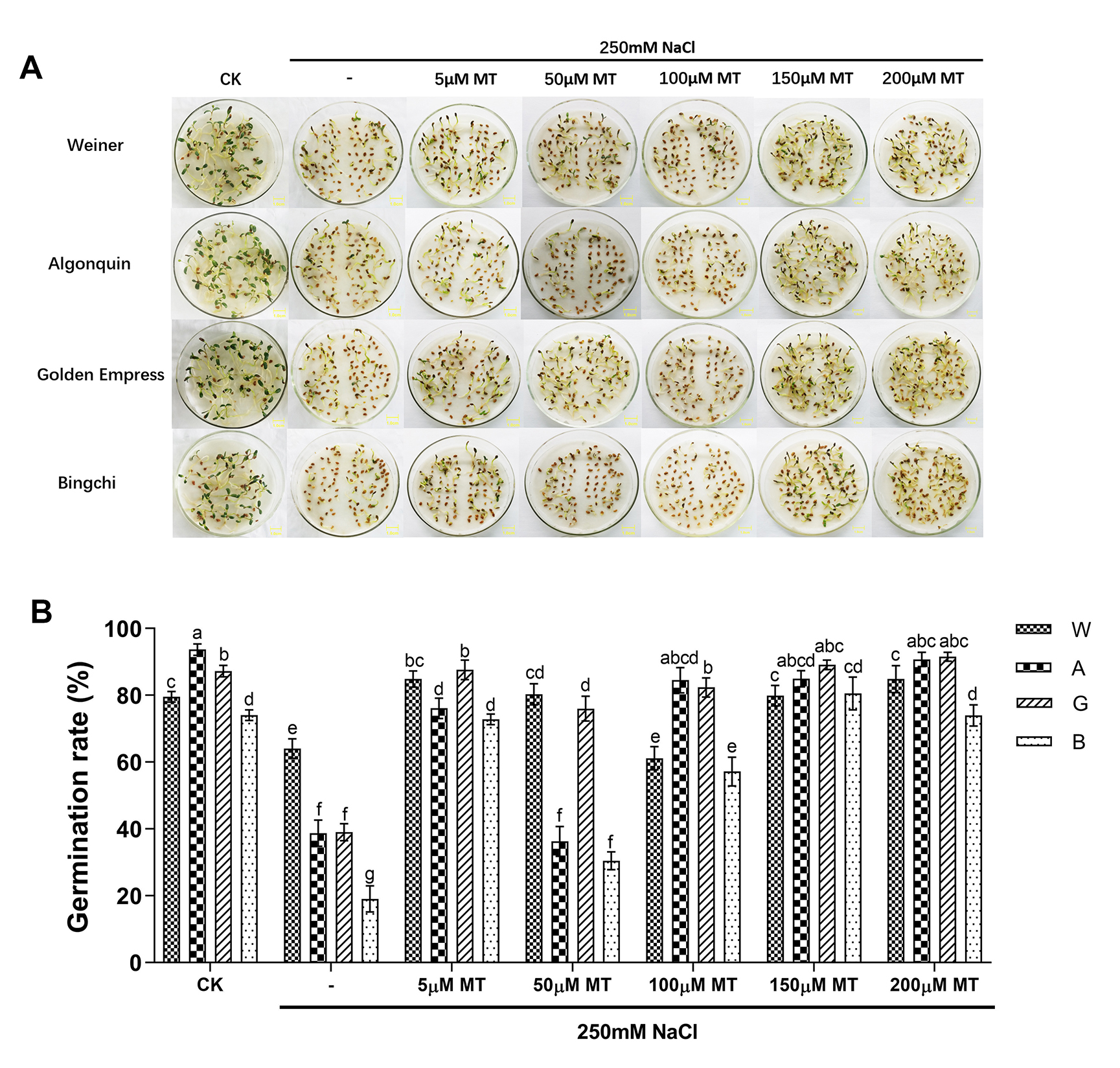

Supplement: Supplementary Figure 3 — Different concentrations of MT treatment. The experiment was carried out as the experiment in Figure 1 unless adding different concentrations of MT. [file Image_3.JPEG]

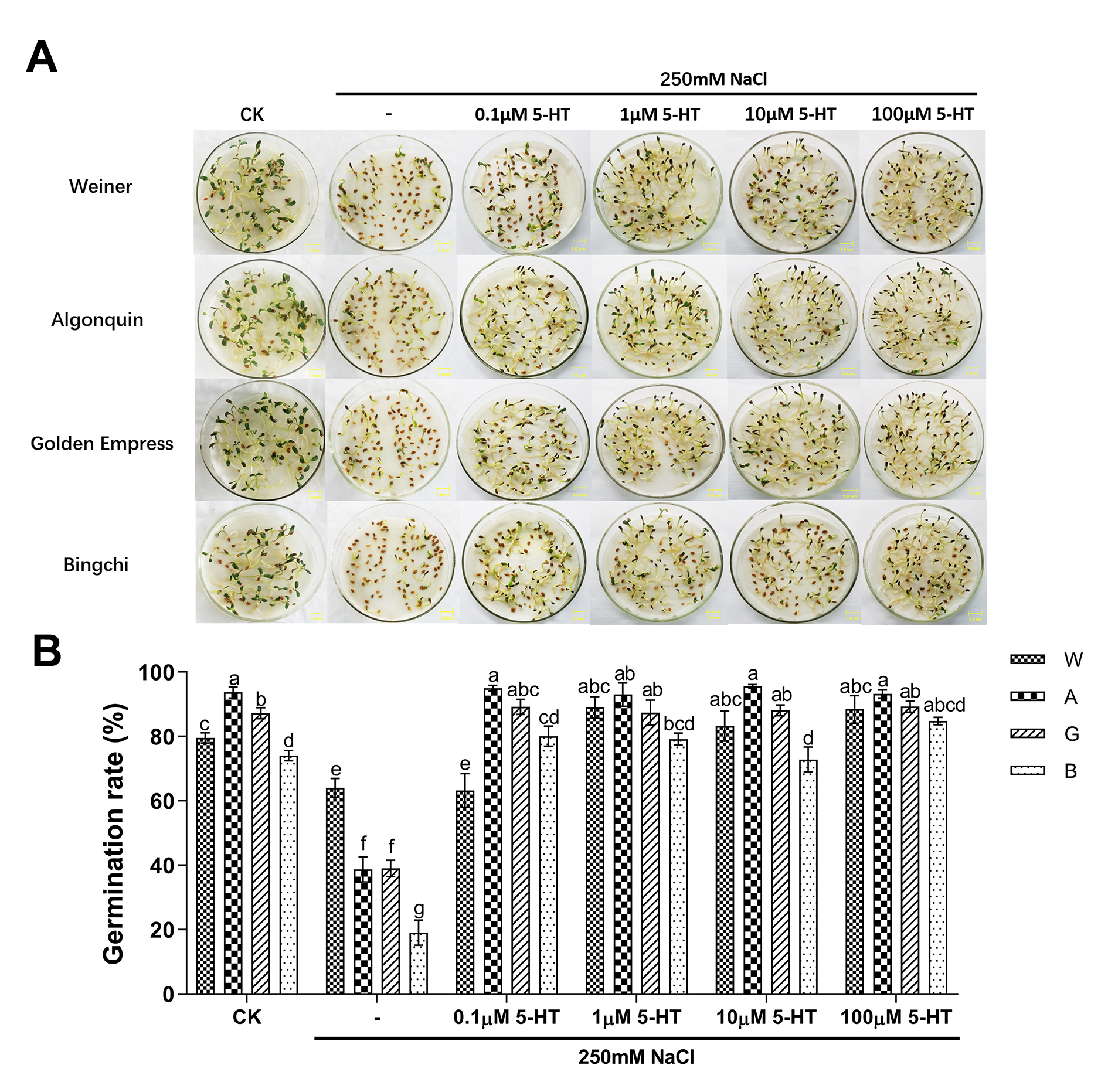

Supplement: Supplementary Figure 4 — Different concentrations of 5-HT treatment. The experiment was carried out as the experiment in Figure 1 unless adding different concentrations of 5-HT. [file Image_4.TIF]

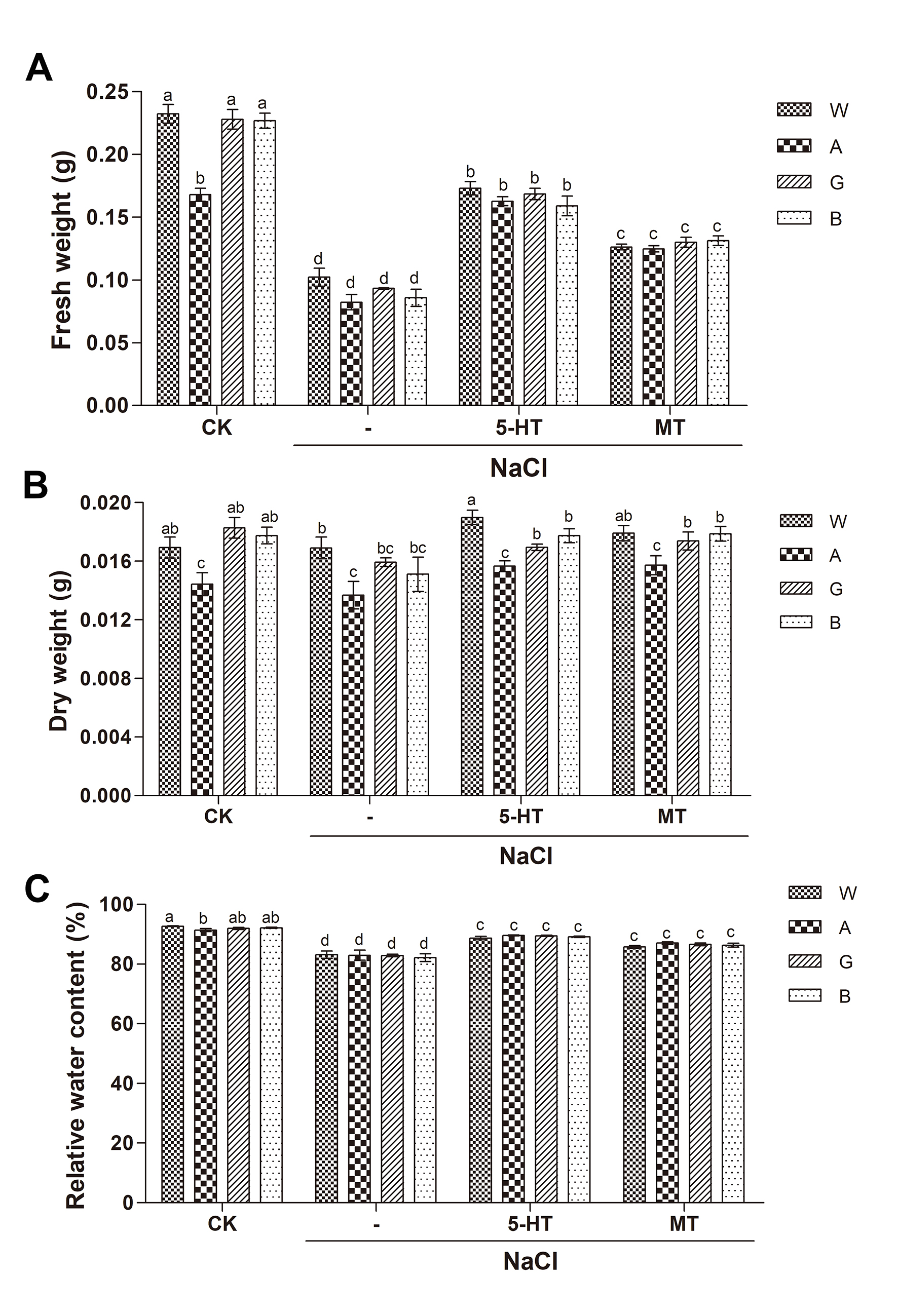

Supplement: Supplementary Figure 5 — Effects of combined treatment on fresh weight (A), dry weight (B), and relative water content (C) of different alfalfa varieties under salt stress. CK: Water. NaCl: 250 mM. MT: 150 μM. 5-HT: 1 μM. Seeds were germinated for 10 days under different treatments, and 30–60 seedlings from each replicate were used for measurement. Water content = [(Fresh weight − Dry weight)/Fresh weight] × 100%. Error bars show the SE between biological replicates and a t-test was performed between different treatment groups. Different numbers indicate significant differences (p ≦ 0.05) between samples. A, Algonquin; B, Bingchi; G, Golden Empress; W, Weiner. [file Image_5.JPEG]

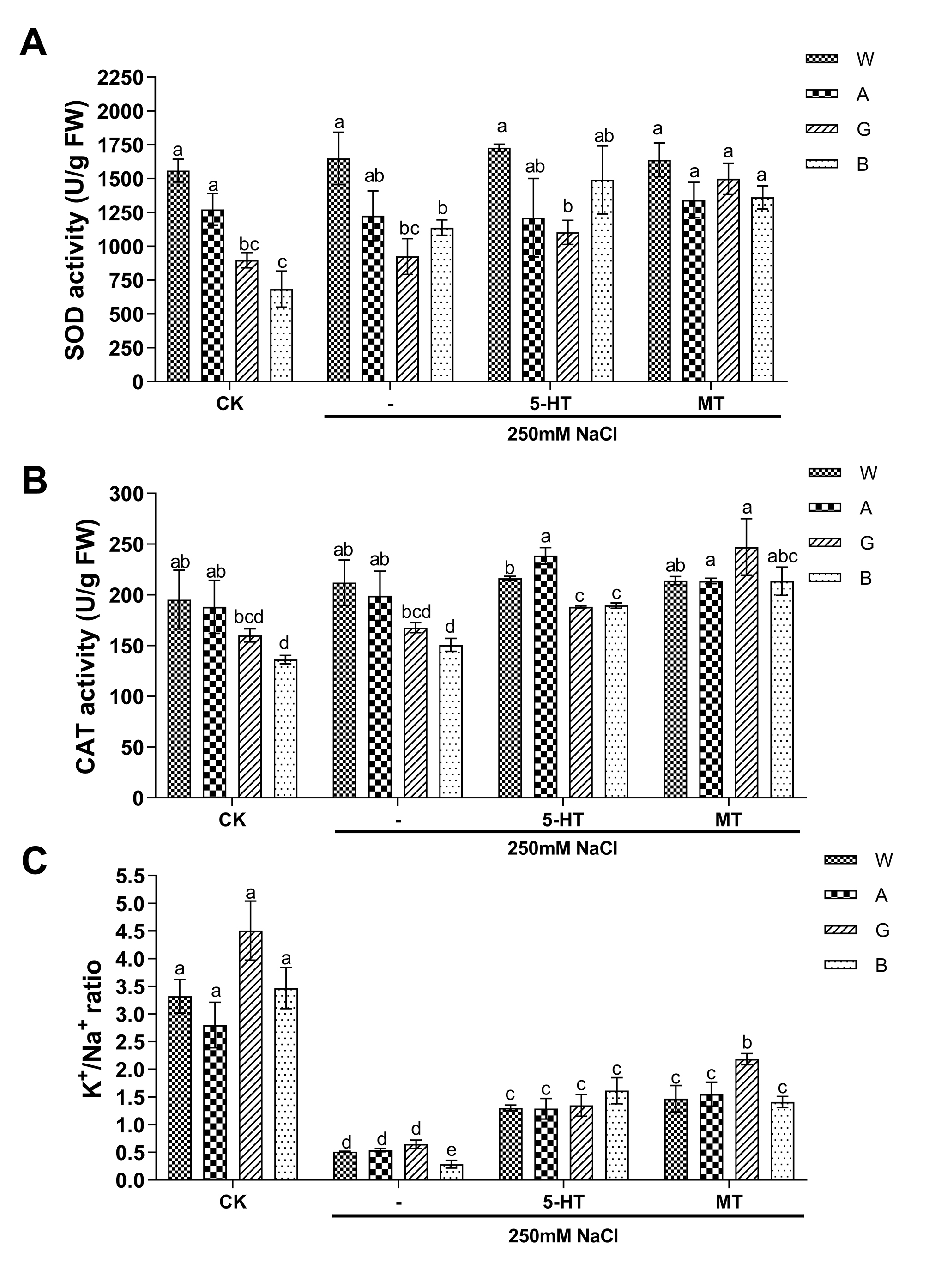

Supplement: Supplementary Figure 6 — Measurement of SOD (A), CAT (B), and K+/Na+ (C). The experiment was carried out as the experiment in Figure 3. [file Image_6.JPEG]

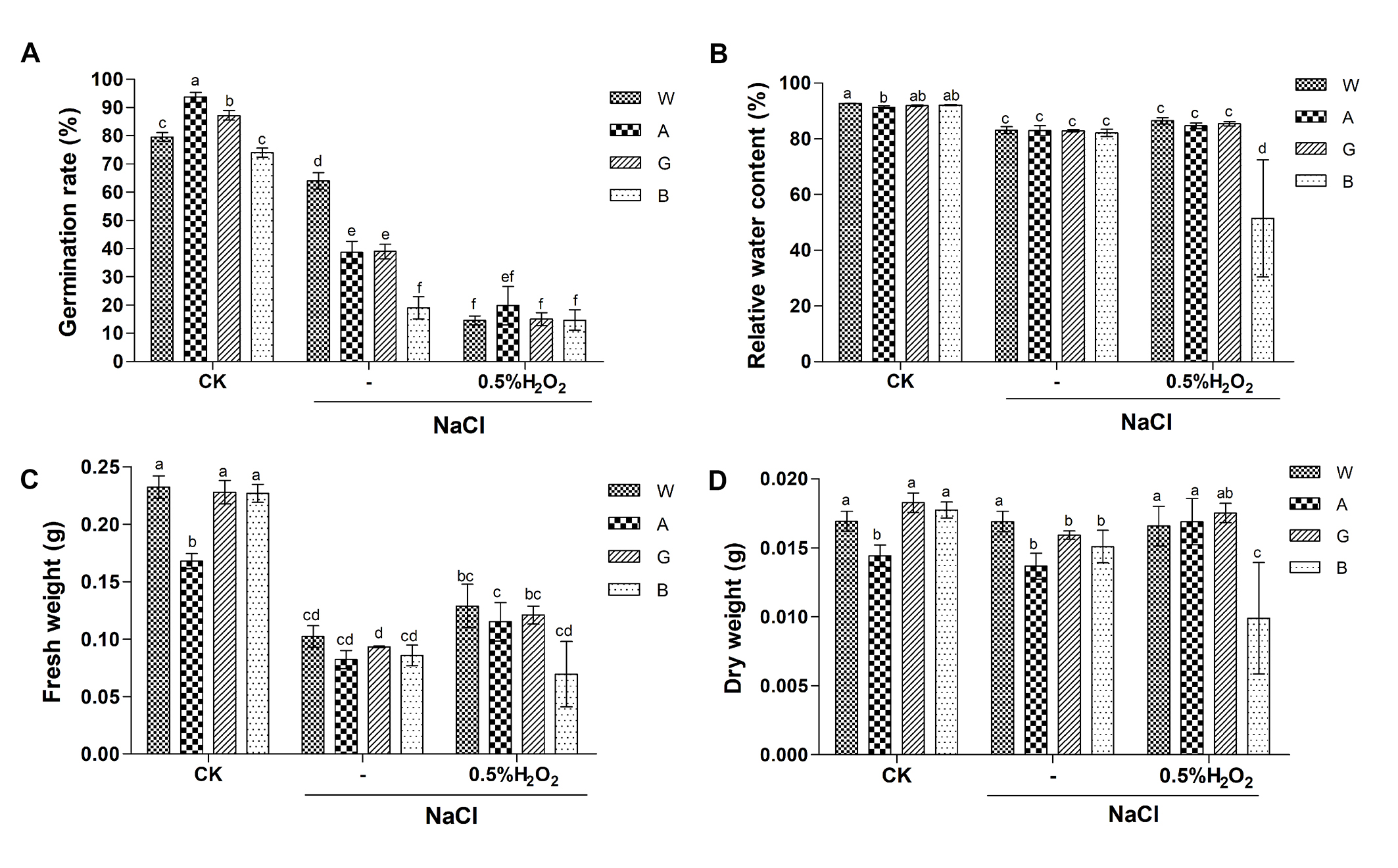

Supplement: Supplementary Figure 7 — Effects of H2O2 treatment on germination and growth of different alfalfa varieties. CK: Water; NaCl: 250 mM. For each replicate, one hundred alfalfa seeds were surface-sterilized, spread evenly on two layers of filter paper which were presoaked with 5 ml treatment solution, and placed in a Petri dish at 20°C in an illuminating incubator. (A) Germination rate. Germinated seeds were calculated on the 10th day and the germination rate was determined. Three biological replicates were performed and error bars show the SE values. (B) Relative water content. (C) Fresh weight. (D) Dry weight. n = 30. A t-test was performed between samples in different treatment groups. Different numbers indicate significant differences (p ≦ 0.05) between different treatments. A, Algonquin; B, Bingchi; G, Golden Empress; W: Weiner. [file Image_7.JPEG]

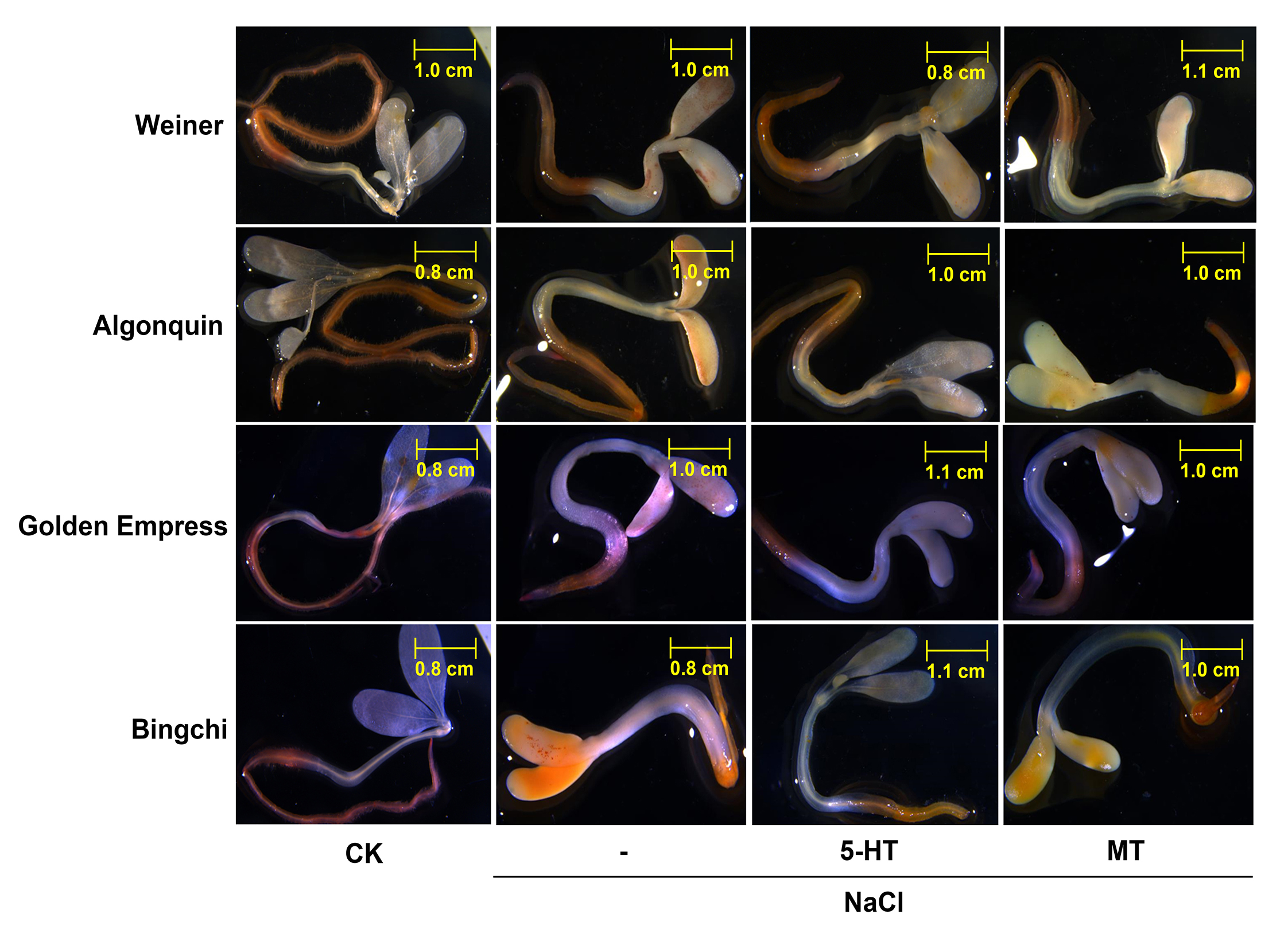

Supplement: Supplementary Figure 8 — DAB-staining of alfalfa seedlings. [file Image_8.JPEG]

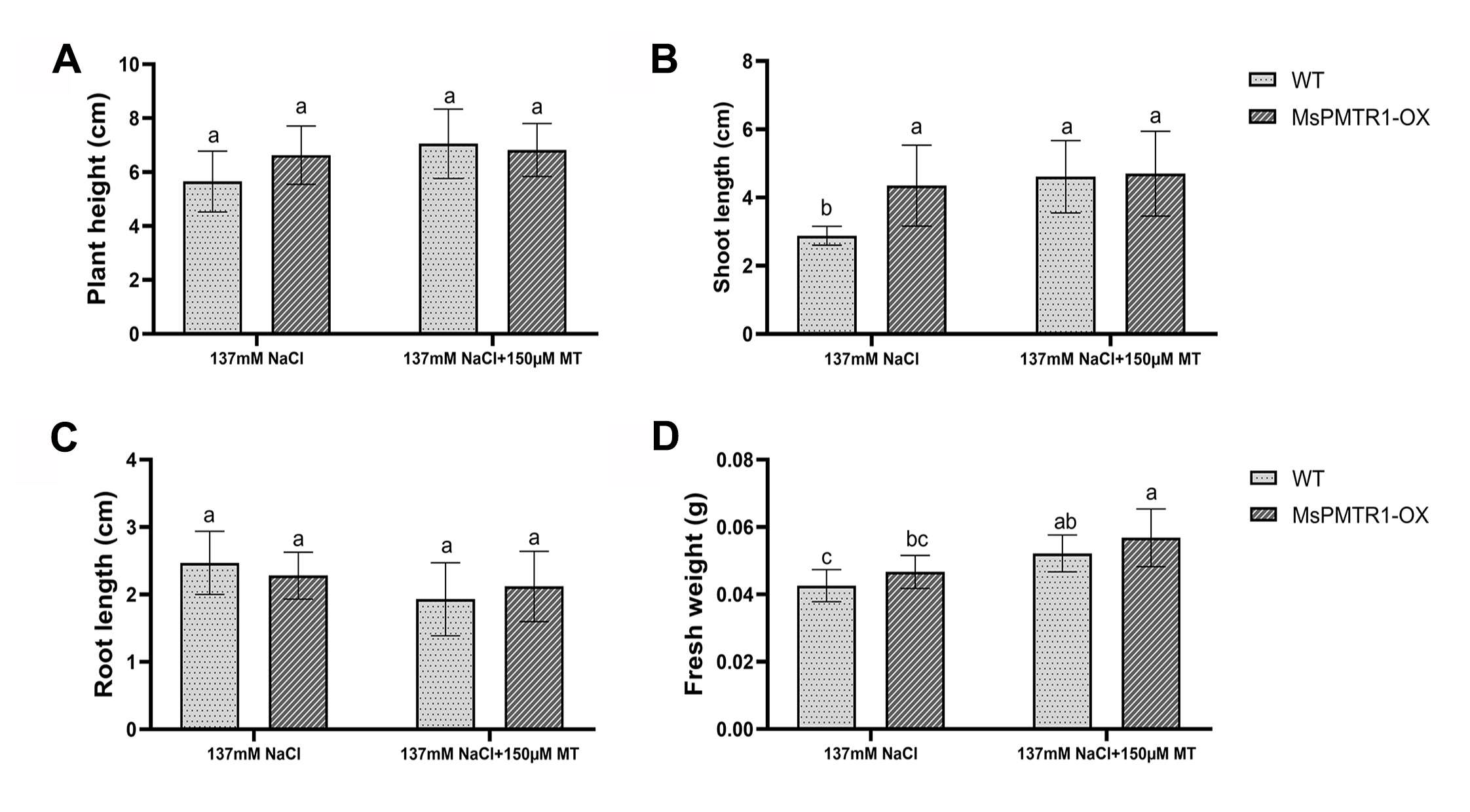

Supplement: Supplementary Figure 9 — Phenotypic observation of another M. truncutula line. The experiment was carried out as the experiment in Figure 9. [file Image_9.JPG]

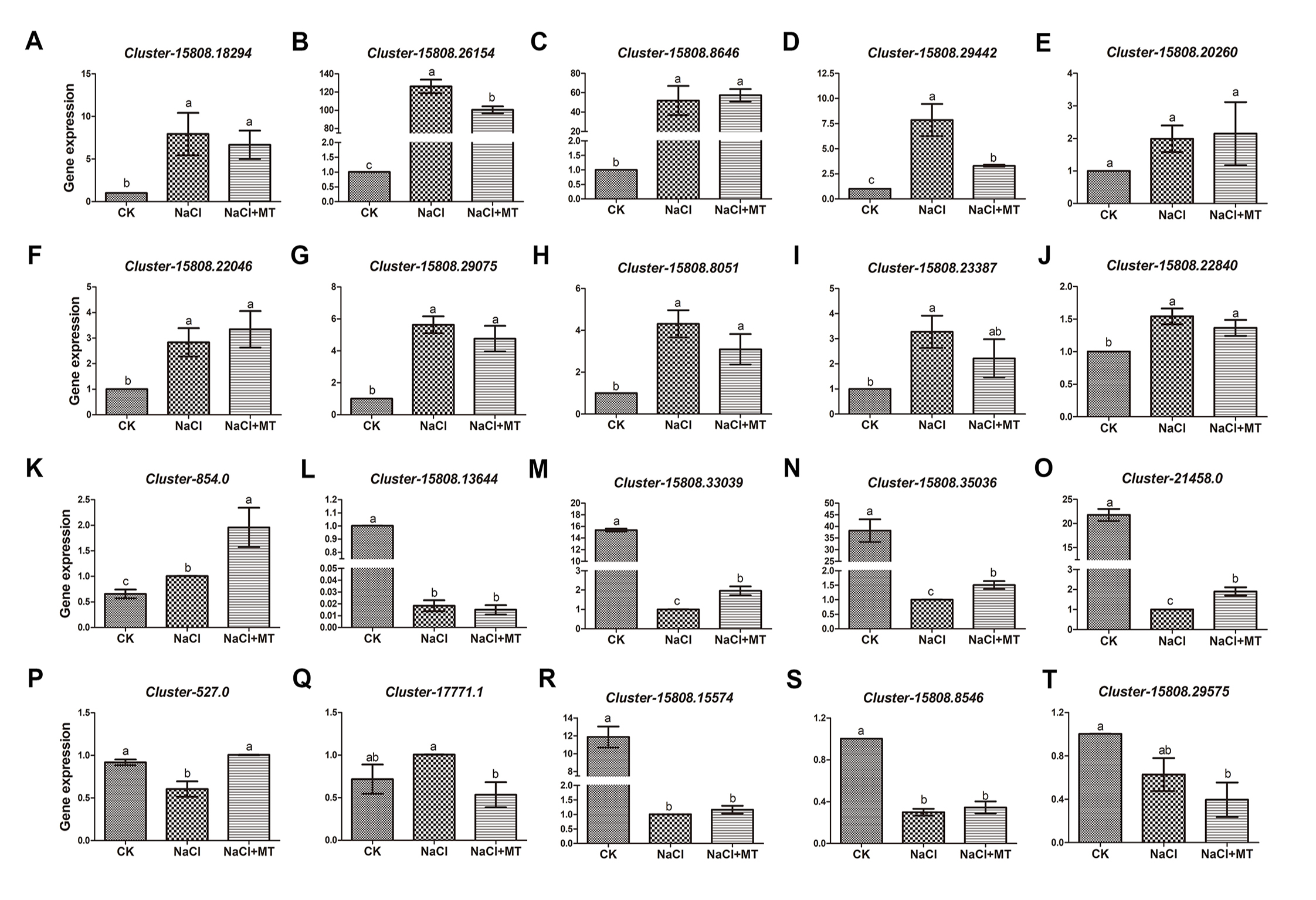

Supplement: Supplementary Figure 10 — The qRT-PCR confirmation of selected unigenes from transcriptome data. The mRNA levels of 20 unigenes were detected by qRT-PCR experiment using gene-specific primers. The housekeeping gene, MsActin was used as the reference. Data represent mean values for three independent biological replicates. SE is indicated by vertical bars. Different numbers indicate significant differences (p ≦ 0.05) between different treatments. CK, Water. NaCl, 250 mM. NaCl + MT, 250 mM NaCl + 150 μM MT. [file Image_10.JPEG]
