# Supplementary material for: Melatonin Enhances Seed Germination and Seedling Growth of Medicago sativa Under Salinity via a Putative Melatonin Receptor MsPMTR1
Source: Front Plant Sci. 2021 Aug 17;12:702875. doi: 10.3389/fpls.2021.702875 (PMC8418131; doi:10.3389/fpls.2021.702875)
Supplement: Supplementary Table 1 — The qRT-PCR primers for the detection of unigene expression. [file Table_1.DOCX]

| q-DEG-1 | Cluster-15808.18294 | 5’-ATCTGGCCACATCTCAGG-3’  5’-ACCCACTTGTGCTTTACACT-3’ | 197bp |
| --- | --- | --- | --- |
| q-DEG-2 | Cluster-15808.26154 | 5’-CGAATCCGAGTCTGAAGGGA-3’  5’-CGCATTCGACAACCACTGAT-3’ | 93bp |
| q-DEG-3 | Cluster-15808.8646 | 5’-GGGATTGGGACTTGCCTAGA-3’  5’-AACTGTGTGCACTCAACCAC-3’ | 151bp |
| q-DEG-4 | Cluster-15808.29442 | 5’-CTCCAAAGCAACTGCAACCT-3’  5’-ATGATCCCAGGGCAAAGGTC-3’ | 95bp |
| q-DEG-5 | Cluster-15808.20260 | 5’-TTGGCACTACGGTACAAGGT-3’  5’-CATTTGGGCGATTGGAAGGT-3’ | 94bp |
| q-DEG-6 | Cluster-15808.22046 | 5’-TGCCAGTACTCAAAGGCAGA-3’  5’-GCTCCGGGTATGACGGATAA-3’ | 156bp |
| q-DEG-7 | Cluster-15808.29075 | 5’-TGCAGAGGAGGCTGGATATG-3’  5’-CTAGAAGCACACCCAAGGGA-3’ | 194bp |
| q-DEG-8 | Cluster-15808.8051 | 5’-TTTCACCGTGCTCTACAGGT-3’  5’-CAGCCAAGCCAAGTATGCAA-3’ | 177bp |
| q-DEG-9 | Cluster-15808.23387 | 5’-CCTAGTCGCAGAGAACCAGT-3’  5’-AGGCAACTGCAGAGTTGTTG -3’ | 103bp |
| q-DEG-10 | Cluster-15808.22840 | 5’-GCTCCTCTGTGAGACCAACT-3’  5’-TTGATGGAGGGAGTGGCATT-3’ | 127bp |
| q-DEG-11 | Cluster-854.0 | 5’-CTCTGCTTGGCCTTGATGAC-3’  5’-GTACTGGCCTGCAACTATGC-3’ | 107bp |
| q-DEG-12 | Cluster-15808.13644 | 5’-AACCCAAATCAGCAGCCAAT-3’  5’-TGTGTTTGGAACCATTCAGACT-3’ | 155bp |
| q-DEG-13 | Cluster-15808.33039 | 5’-GAGTGGTGGCAGGTATAGGG-3’  5’-CGTGCAGGGAGGAACTTAGA-3’ | 199bp |
| q-DEG-14 | Cluster-15808.35036 | 5’-GTCTTGGTACTAGGGTGCGT-3’  5’-TCCACACTGGCCTTGATTCT-3’ | 128bp |
| q-DEG-15 | Cluster-21458.0 | 5’-CTTTGGCCACATACATGCGA-3’  5’-CCGCACATTGATCCTCTTGG-3’ | 200bp |
| q-DEG-16 | Cluster-527.0 | 5’-GGGACGAACCTCAAGGATGA-3’  5’-AGGACTAGCAAAGGCTGGAA-3’ | 165bp |
| q-DEG-17 | Cluster-17771.1 | 5’-TGTGTGCTAGACCCTCCAAG-3’  5’-AGGGAGAGCGCAAGTTACAT-3’ | 115bp |
| q-DEG-18 | Cluster-15808.15574 | 5’-ATGTTCTCCCTTGGTCATGT-3’  5’-TCCTCTTGTCCAATGCTACT-3’ | 191bp |
| q-DEG-19 | Cluster-15808.8546 | 5’-GGGTTCACAGACGGAATTGG-3’  5’-TTTGCACTGCCTGCATTTCT-3’ | 161bp |
| q-DEG-20 | Cluster-15808.29575 | 5’-TCCCAGCTGTTTCTCCAACA-3’  5’-GGATAGATCTCCGTTGTTGCC-3’ | 187bp |
| q-MsActin | JQ028730.1 | 5’-CCGACCTCGTCATACTGGTG-3’  5’-TCTTCAGGAGCAACACGCAA-3’ | 192b |

**Table S1，**qRT-PCR primers for detection of unigene expression.

**Table S2**, Statistical output of RNA-seq data for selected DEGs in Figure 10.

| **DEGs with similar expression patterns in NaCl and NaCl+MT groups compared with CK** | | | | | | | | |
| --- | --- | --- | --- | --- | --- | --- | --- | --- |
| **gene_id** | **KO Name** | **KO Description** | **NaCl+MT vsCK** | **log2FC (NaCl+MT vsCK)** | **NaCl+MT vsNaCl** | **log2FC (NaCl+MT vsNaCl)** | **NaClvsCK** | **log2FC (NaClvsCK)** |
| **Glycan degradation** | | | | | | | | |
| Cluster-15808.18294 | GLB1, ELNR1 | beta-galactosidase | UP | 1.0525 | FALSE | -0.04532 | UP | 1.1185 |
| Cluster-15808.19306 | E3.2.1.24 | alpha-mannosidase | UP | 0.95051 | FALSE | -0.069785 | UP | 1.041 |
| Cluster-15808.30709 | AXY8, FUC95A, afcA | alpha-L-fucosidase 2 | UP | 2.2545 | FALSE | 0.1297 | UP | 2.1464 |
| Cluster-15808.19307 | E3.2.1.24 | alpha-mannosidase | UP | 0.88824 | FALSE | -0.091321 | UP | 1.0003 |
| Cluster-15808.11537 | E3.2.1.96 | Glycosyl hydrolase family | UP | 0.80052 | FALSE | 0.076297 | UP | 0.74746 |
| **Glyoxylate and dicarboxylate metabolism** | | | | | | | | |
| Cluster-15808.26154 | E4.1.3.1, aceA | isocitrate lyase | UP | 4.5132 | FALSE | -0.24212 | UP | 4.7776 |
| Cluster-15808.26155 | E4.1.3.1, aceA | isocitrate lyase | UP | 4.4077 | FALSE | -0.19166 | UP | 4.6217 |
| Cluster-15808.19204 | rbcS | ribulose-bisphosphate carboxylase | UP | 0.91009 | FALSE | 0.098323 | UP | 0.83347 |
| Cluster-15808.8646 | E4.1.3.1, aceA | isocitrate lyase | UP | 4.4772 | FALSE | -0.087846 | UP | 4.5955 |
| Cluster-15808.9989 | E2.3.1.9, atoB | acetyl-CoA C-acetyltransferase | UP | 1.4596 | FALSE | 0.017964 | UP | 1.4638 |
| Cluster-15808.12606 | GLYR | glyoxylate | UP | 1.3159 | FALSE | -0.089363 | UP | 1.4275 |
| **Citrate cycle (TCA cycle)** | | | | | | | | |
| Cluster-15808.23242 | MDH1 | malate dehydrogenase | UP | 0.73358 | FALSE | -0.017934 | UP | 0.77548 |
| Cluster-15808.12055 | MDH2 | malate dehydrogenase | UP | 0.90272 | FALSE | -0.18466 | UP | 1.1106 |
| Cluster-15808.29443 | CS, gltA | citrate synthase | UP | 3.3108 | FALSE | -0.36027 | UP | 3.6944 |
| Cluster-15808.29576 | IDH1, IDH2, icd | isocitrate dehydrogenase | UP | 0.53095 | FALSE | -0.03556 | UP | 0.58829 |
| Cluster-15808.29442 | CS, gltA | citrate synthase | UP | 3.8587 | FALSE | -0.29945 | UP | 4.1806 |
| Cluster-15808.29438 | CS, gltA | citrate synthase | UP | 0.61074 | FALSE | -0.019366 | UP | 0.65319 |
| Cluster-15808.20650 | E4.1.1.49, pckA | phosphoenolpyruvate carboxykinase | UP | 0.79323 | FALSE | -0.1829 | UP | 0.99913 |
| Cluster-15808.20891 | E4.1.1.49, pckA | phosphoenolpyruvate carboxykinase | UP | 0.88606 | FALSE | -0.14926 | UP | 1.0586 |
| Cluster-15808.19608 | ACO, acnA | aconitate hydratase | UP | 0.75678 | FALSE | -0.050743 | UP | 0.83033 |
| **Amylase** | | | | | | | | |
| Cluster-15808.17855 | E3.2.1.2 | beta-amylase | UP | 0.64539 | FALSE | -0.044347 | UP | 0.71291 |
| Cluster-15808.25479 | E3.2.1.2 | beta-amylase | UP | 1.3338 | FALSE | 0.44473 | FALSE | 0.90646 |
| Cluster-15808.20260 | E3.2.1.2 | beta-amylase | UP | 1.6916 | FALSE | 0.016747 | UP | 1.6981 |
| Cluster-15808.18362 | XYL1 | alpha-D-xyloside xylohydrolase | UP | 2.1476 | FALSE | 0.038818 | UP | 2.1311 |
| Cluster-15808.28555 | E3.2.1.2 | beta-amylase | UP | 1.6387 | FALSE | -0.065125 | UP | 1.7273 |
| Cluster-15808.22046 | E3.2.1.2 | beta-amylase | UP | 1.9859 | FALSE | -0.025681 | UP | 2.0352 |
| Cluster-15808.33260 | glgB | Alpha amylase | UP | 1.3751 | FALSE | 0.24181 | UP | 1.1533 |
| Cluster-15808.17165 | XYL1 | alpha-D-xyloside xylohydrolase | UP | 2.1641 | FALSE | 0.010275 | UP | 2.1762 |
| Cluster-1309.0 | glgB | 1,4-alpha-glucan branching enzyme | UP | 4.6884 | FALSE | 2.2043 | FALSE | 2.4559 |
| **Proline biosynthesis** | | | | | | | | |
| Cluster-15808.29075 | ALDH18A1, P5CS | delta-1-pyrroline-5-carboxylate synthetase | UP | 2.3021 | FALSE | 0.09021 | UP | 2.2335 |
| Cluster-15808.29410 | E1.2.1.88 | 1-pyrroline-5-carboxylate dehydrogenase | UP | 1.3464 | FALSE | -0.045454 | UP | 1.4143 |
| Cluster-15808.29082 | ALDH18A1, P5CS | delta-1-pyrroline-5-carboxylate synthetase | UP | 2.1494 | FALSE | 0.0051549 | UP | 2.1667 |
| Cluster-15808.29081 | ALDH18A1, P5CS | delta-1-pyrroline-5-carboxylate synthetase | UP | 2.1415 | FALSE | 0.091242 | UP | 2.0731 |
| Cluster-15808.14899 | proC | pyrroline-5-carboxylate reductase | UP | 1.166 | FALSE | 0.0058898 | UP | 1.1834 |
| Cluster-15808.8051 | E1.14.11.2 | prolyl 4-hydroxylase | UP | 4.0239 | FALSE | -0.55434 | UP | 4.5995 |
| Cluster-15808.29080 | ALDH18A1, P5CS | delta-1-pyrroline-5-carboxylate synthetase | UP | 2.1381 | FALSE | 0.10973 | UP | 2.0501 |
| Cluster-15808.29078 | ALDH18A1, P5CS | delta-1-pyrroline-5-carboxylate synthetase | UP | 2.161 | FALSE | 0.14114 | UP | 2.0423 |
| Cluster-15808.29076 | ALDH18A1, P5CS | delta-1-pyrroline-5-carboxylate synthetase | UP | 2.0157 | FALSE | -0.02134 | UP | 2.0589 |
| Cluster-15808.29077 | ALDH18A1, P5CS | delta-1-pyrroline-5-carboxylate synthetase | UP | 2.1452 | FALSE | 0.023809 | UP | 2.1438 |
| Cluster-15808.14234 | ALDH18A1, P5CS | delta-1-pyrroline-5-carboxylate synthetase | UP | 0.75348 | FALSE | -0.11718 | UP | 0.89275 |
| **Proline degradation** | | | | | | | | |
| Cluster-15808.34801 | PRODH | proline dehydrogenase | DOWN | -3.3442 | FALSE | -0.21315 | DOWN | -3.1085 |
| Cluster-15808.13644 | PRODH | proline dehydrogenase | DOWN | -4.6173 | FALSE | -0.24327 | DOWN | -4.3506 |
| Cluster-15808.13642 | PRODH | proline dehydrogenase | DOWN | -4.4187 | FALSE | -0.087325 | DOWN | -4.3081 |
| Cluster-15808.13643 | PRODH | proline dehydrogenase | DOWN | -3.4822 | FALSE | 0.089976 | DOWN | -3.5483 |
| **Polyamine biosynthesis** | | | | | | | | |
| Cluster-15808.23387 | ASP5 | aspartate aminotransferase | UP | 1.1515 | FALSE | -0.19664 | UP | 1.3714 |
| Cluster-15808.20977 | PAO4, PAO3, PAO2 | polyamine oxidase | UP | 1.5633 | FALSE | 0.0099847 | UP | 1.5769 |
| Cluster-15808.19289 | speE, SRM | spermidine synthase | UP | 0.66167 | FALSE | 0.045345 | UP | 0.64016 |
| Cluster-15808.14035 | GOT2 | aspartate aminotransferase | UP | 1.1365 | FALSE | -0.19653 | UP | 1.3559 |
| Cluster-15808.23668 | GOT1 | aspartate aminotransferase | FALSE | 0.5541 | FALSE | -0.071837 | UP | 0.65003 |
| Cluster-15808.17588 | speD, AMD1 | S-adenosylmethionine decarboxylase | UP | 0.93431 | FALSE | -0.012269 | UP | 0.96843 |
| Cluster-15808.8546 | speE, SRM | spermidine synthase | UP | 1.1993 | FALSE | 0.025181 | UP | 1.1968 |
| Cluster-15808.28934 | ALDH7A1 | aldehyde dehydrogenase | UP | 1.6428 | FALSE | -0.02818 | UP | 1.6935 |
| Cluster-15808.32646 | E1.2.1.3 | aldehyde dehydrogenase (NAD+) | UP | 1.6577 | FALSE | -0.0089515 | UP | 1.6885 |
| Cluster-15808.26996 | E3.5.3.1, rocF, arg | arginase | UP | 1.0564 | FALSE | 0.00090875 | UP | 1.0791 |
| Cluster-15808.17592 | speD, AMD1 | S-adenosylmethionine decarboxylase | UP | 0.87237 | FALSE | -0.044317 | UP | 0.93816 |
| **Glutathione metabolism** | | | | | | | | |
| Cluster-15808.22840 | GSR | glutathione reductase (NADPH) | UP | 0.9841 | FALSE | -0.010652 | UP | 1.0175 |
| Cluster-15808.29575 | GST | glutathione S-transferase | UP | 1.2585 | FALSE | 0.0034034 | UP | 1.2749 |
| Cluster-15808.27867 | GST | glutathione S-transferase | UP | 1.0492 | FALSE | 0.17287 | UP | 0.89596 |
| Cluster-15808.33075 | GST | glutathione S-transferase | UP | 0.90141 | FALSE | 0.052085 | UP | 0.87474 |
| Cluster-15808.35005 | GST | glutathione S-transferase | UP | 1.1244 | FALSE | 0.23993 | UP | 0.90368 |
| **The up-regulated genes in NaCl+MT compared with NaCl** | | | | | | | | |
| **gene_id** | **KO Name** | **KO Description** | **NaCl+MT vsCK** | **log2FC (NaCl+MT vsCK)** | **NaCl+MT vsNaCl** | **log2FC (NaCl+MT vsNaCl)** | **NaClvsCK** | **log2FC (NaClvsCK)** |
| **Ribosome,elongation factor** | | | | | | | | |
| Cluster-16461.0 | RP-S3e, RPS3 | small subunit ribosomal protein S3e | FALSE | 0.65849 | UP | 3.553 | DOWN | -2.87 |
| Cluster-19907.0 | RP-L13e, RPL13 | large subunit ribosomal protein L13e | FALSE | 0.5377 | UP | 3.2895 | FALSE | -2.7095 |
| Cluster-10172.0 | RP-S30e, RPS30 | small subunit ribosomal protein S30e | FALSE | 0.04915 | UP | 5.1402 | DOWN | -5.0671 |
| Cluster-11417.0 | RP-S23e, RPS23 | small subunit ribosomal protein S23e | FALSE | -0.87387 | UP | 4.9914 | DOWN | -5.8424 |
| Cluster-13483.0 | RP-S5e, RPS5 | small subunit ribosomal protein S5e | FALSE | 1.4351 | UP | 4.5354 | FALSE | -3.0726 |
| Cluster-5970.1 | RP-L3e, RPL3 | large subunit ribosomal protein L3e | FALSE | 1.0382 | UP | 4.7068 | FALSE | -3.6469 |
| Cluster-8556.0 | RP-S9e, RPS9 | small subunit ribosomal protein S9e | FALSE | 0.84686 | UP | 3.1885 | FALSE | -2.3155 |
| Cluster-19870.0 | RP-S24e, RPS24 | small subunit ribosomal protein S24e | FALSE | 1.7991 | UP | 4.2864 | FALSE | -2.4706 |
| Cluster-12212.0 | RP-L15e, RPL15 | large subunit ribosomal protein L15e | FALSE | 0.13181 | UP | 3.1551 | DOWN | -3.0016 |
| Cluster-21458.0 | RP-L21e, RPL21 | large subunit ribosomal protein L21e | FALSE | -1.6778 | UP | 4.8546 | DOWN | -6.5078 |
| Cluster-15225.0 | RP-S3Ae, RPS3A | small subunit ribosomal protein S3Ae | FALSE | 0.46468 | UP | 3.3236 | DOWN | -2.8382 |
| Cluster-15808.18503 | EEF1A | elongation factor 1-alpha | FALSE | -0.088449 | UP | 0.90671 | DOWN | -0.97244 |
| Cluster-16882.0 | RP-L7e, RPL7 | large subunit ribosomal protein L7e | FALSE | -0.006025 | UP | 3.9697 | DOWN | -3.9637 |
| Cluster-17126.0 | RP-L14e, RPL14 | large subunit ribosomal protein L14e | FALSE | 1.0768 | UP | 3.1953 | FALSE | -2.0628 |
| Cluster-19808.0 | RP-L37Ae, RPL37A | large subunit ribosomal protein L37Ae | FALSE | -0.092714 | UP | 4.9039 | DOWN | -4.9737 |
| Cluster-17483.0 | RP-L9e, RPL9 | large subunit ribosomal protein L9e | FALSE | 0.62077 | UP | 4.7918 | DOWN | -4.1475 |
| Cluster-10478.0 | RP-L32e, RPL32 | large subunit ribosomal protein L32e | FALSE | 0.089681 | UP | 4.1121 | DOWN | -3.9974 |
| **Membrane integrity** | | | | | | | | |
| Cluster-15808.24419 | WAT1 | integral component of membrane | FALSE | -0.53726 | UP | 0.82516 | DOWN | -1.3414 |
| Cluster-10309.0 | -- | integral component of membrane | UP | 1.9937 | UP | 1.4145 | FALSE | 0.59713 |
| Cluster-15808.20713 | -- | cell wall | DOWN | -1.3626 | UP | 0.85114 | DOWN | -2.187 |
| Cluster-15808.15548 | -- | integral component of membrane | FALSE | 0.84439 | UP | 0.69941 | FALSE | 0.16716 |
| Cluster-15808.15574 | -- | integral component of membrane | FALSE | -0.777 | UP | 3.6953 | DOWN | -4.4421 |
| **photosynthesis** | | | | | | | | |
| Cluster-854.0 | psaN | photosystem I subunit PsaN | UP | 6.4227 | UP | 1.6942 | UP | 4.7437 |
| Cluster-20807.0 | PsbL protein | photosystem II | FALSE | -0.61713 | UP | 0.74955 | DOWN | -1.3455 |
| **Transporter** | | | | | | | | |
| Cluster-15808.26731 | -- | chloride transport | FALSE | -0.61861 | UP | 0.51393 | DOWN | -1.1108 |
| Cluster-20798.0 | -- | metal ion binding | FALSE | -2.148 | UP | 4.8161 | DOWN | -6.9237 |
| Cluster-105.0 | -- | amino acid permease | FALSE | 4.4999 | UP | 4.7844 | FALSE | NA |
| Cluster-527.0 | -- | ABC transporter | UP | 4.7043 | UP | 4.9851 | FALSE | NA |
| Cluster-15808.720 | -- | proton-transporting V-type ATPase | FALSE | -0.27767 | UP | 0.97431 | DOWN | -1.2298 |
| Cluster-5452.0 | TIP | aquaporin TIP | UP | 2.8007 | UP | 0.73448 | UP | 2.0928 |
| Cluster-15808.35044 | -- | ion transmembrane transport | FALSE | 1.6565 | UP | 4.088 | FALSE | -2.3815 |
| Cluster-19257.0 | SLC2A13, ITR | Sugar (and other) transporter | DOWN | -1.238 | UP | 0.80071 | DOWN | -2.0175 |
| **Heat shock protein** | | | | | | | | |
| Cluster-11670.0 | HSPA1_8 | heat shock 70kDa protein | FALSE | -0.11754 | UP | 1.1744 | DOWN | -1.2699 |
| Cluster-15808.26508 | groEL, HSPD1 | chaperonin GroEL | UP | 3.1039 | UP | 1.4462 | FALSE | 1.668 |
| Cluster-15808.11668 | HSP70 | Heat shock cognate 70 kDa protein | FALSE | -0.53637 | UP | 2.736 | DOWN | -3.2422 |
| Cluster-15808.13012 | HSFF | Heat shock factor protein HSF30 | UP | 2.7508 | UP | 1.0446 | UP | 1.7304 |
| **Stress response** | | | | | | | | |
| Cluster-15808.27484 | CRT..DRE | Dehydration-responsive element | FALSE | -0.1624 | UP | 0.69604 | FALSE | -0.83312 |
| Cluster-15808.34358 | MYBP | Transcription factor MYB2 | UP | 3.2125 | UP | 1.2219 | UP | 2.0167 |
| Cluster-15808.34817 | SLIDE | Zinc finger BED domain | UP | 3.0812 | UP | 1.4759 | FALSE | 1.6127 |
| Cluster-15808.31461 | SWIB/MDM2 domain | Zinc finger CCCH domain | UP | 1.5323 | UP | 0.91 | FALSE | 0.64475 |
| Cluster-15808.891 | -- | Remorin | FALSE | 0.50212 | UP | 0.70494 | FALSE | -0.18389 |
| Cluster-15808.5521 | -- | HD-ZIP protein | FALSE | 1.0149 | UP | 0.89539 | FALSE | 0.14782 |
| Cluster-15808.8353 | -- | Transcription repressor OFP4 | FALSE | 0.25156 | UP | 1.1404 | FALSE | -0.8707 |
| Cluster-15808.5853 | -- | defense response | UP | 2.6875 | UP | 0.52531 | UP | 2.1866 |
| Cluster-15808.33039 | AP2 domain | Dehydration-responsive element | FALSE | 1.1909 | UP | 1.3331 | FALSE | -0.10513 |
| Cluster-15808.23974 | SAUR | SAUR family protein | FALSE | -0.99915 | UP | 1.0325 | DOWN | -2.0129 |
| **Peroxidase and oxidoreductase** | | | | | | | | |
| Cluster-21379.0 | E1.11.1.7 | peroxidase | FALSE | -0.38694 | UP | 0.73242 | DOWN | -1.0991 |
| Cluster-2216.0 | E1.11.1.7 | peroxidase | UP | 3.323 | UP | 2.9508 | FALSE | 0.46992 |
| Cluster-15808.32831 | E1.11.1.7 | peroxidase | UP | 1.3687 | FALSE | 0.44308 | UP | 0.94474 |
| Cluster-15808.2257 | E1.11.1.7 | peroxidase | UP | 3.8573 | FALSE | 0.31986 | FALSE | 3.5615 |
| Cluster-15808.5838 | E1.11.1.7 | peroxidase | UP | 0.53079 | FALSE | 0.20962 | FALSE | 0.34275 |
| Cluster-15808.34897 | E1.11.1.7 | peroxidase | UP | 0.96496 | FALSE | 0.097264 | UP | 0.89034 |
| Cluster-15808.5614 | E1.11.1.7 | peroxidase | UP | 1.2868 | FALSE | 0.47306 | UP | 0.83493 |
| Cluster-15808.26254 | E1.11.1.7 | peroxidase | UP | 0.61466 | FALSE | 0.17269 | FALSE | 0.46532 |
| Cluster-15808.34865 | -- | 2OG-Fe(II) oxygenase superfamily | UP | 4.4286 | UP | 4.7136 | FALSE | NA |
| Cluster-10611.0 | -- | Cytochrome c oxidase subunit IV | FALSE | -0.035256 | UP | 3.4817 | DOWN | -3.4963 |
| Cluster-15808.4577 | -- | 2OG-Fe(II) oxygenase superfamily | UP | 2.8925 | UP | 1.2033 | FALSE | 1.7059 |
| **Protease** | | | | | | | | |
| Cluster-15808.12669 | -- | Aspartyl protease | FALSE | 0.52788 | UP | 2.0054 | FALSE | -1.4632 |
| Cluster-15808.35036 | papain | cysteine-type endopeptidase | FALSE | 0.76897 | UP | 4.6867 | FALSE | -3.8973 |
| Cluster-17267.0 | Ulp1 protease family, C-terminal catalytic domain | cysteine-type peptidase | FALSE | 0.19276 | UP | 1.4846 | FALSE | -1.2712 |
| Cluster-12075.2 | Caspase domain | cysteine-type endopeptidase | FALSE | -0.74819 | UP | 3.4019 | DOWN | -4.1564 |
| **Sugar and lipid metabolism** | | | | | | | | |
| Cluster-13503.0 | PGK, pgk | phosphoglycerate kinase | FALSE | -0.69742 | UP | 3.623 | DOWN | -4.2921 |
| Cluster-15808.17279 | E2.4.1.46 | beta-galactosyltransferase | DOWN | -1.3635 | UP | 0.68193 | DOWN | -2.0238 |
| Cluster-15808.14809 | UGT74B1 | beta-glucosyltransferase | FALSE | -3.1908 | UP | 4.3283 | DOWN | -7.4919 |
| Cluster-844.0 | -- | Glycosyl hydrolases | UP | 4.4326 | UP | 3.754 | FALSE | 0.69978 |
| Cluster-9605.0 | -- | UDP-Glycosyltransferase | DOWN | -2.2794 | UP | 4.895 | DOWN | -7.1288 |
| Cluster-15808.6513 | -- | serine family amino acid metabolic process | FALSE | 1.0063 | UP | 1.0999 | FALSE | -0.070316 |
| **The down-regulated genes in NaCl+MT compared with NaCl** | | | | | | | | |
| **gene_id** | **KO Name** | **KO Description** | **NaCl+MT vsCK** | **log2FC (NaCl+MT vsCK)** | **NaCl+MT vsNaCl** | **log2FC (NaCl+MT vsNaCl)** | **NaClvsCK** | **log2FC (NaClvsCK)** |
| **Transcription factors** | | | | | | | | |
| Cluster-15808.16416 | -- | Ethylene-responsive transcription factor ERF017 | DOWN | -2.42 | DOWN | -1.0004 | DOWN | -1.398 |
| Cluster-15808.16414 | -- | Ethylene-responsive transcription factor ERF017 | DOWN | -2.579 | DOWN | -0.7278 | DOWN | -1.8263 |
| Cluster-15808.21513 | -- | zinc finger CCCH domain protein | DOWN | -2.7942 | DOWN | -1.0156 | DOWN | -1.7548 |
| Cluster-15808.12343 | EREBP | Ethylene-responsive transcription factor RAP2-3 | UP | 1.9375 | DOWN | -0.99787 | UP | 2.9582 |
| Cluster-13036.0 | SAUR | Auxin-responsive protein SAUR23 | FALSE | -0.28428 | DOWN | -1.5569 | FALSE | 1.2893 |
| Cluster-15808.16270 | -- | Dof domain, zinc finger | DOWN | -2.8552 | DOWN | -3.9352 | FALSE | 1.1018 |
| Cluster-6984.0 | NPR1 | Ankyrin repeat//BTB/ | UP | 4.3036 | DOWN | -0.90914 | UP | 5.2291 |
| Cluster-12876.1 | -- | CW-type Zinc Finger | FALSE | -2.1039 | DOWN | -4.6411 | FALSE | 2.5148 |
| **Seed linoleate and 9S-lipoxygenase** | | | | | | | | |
| Cluster-17771.1 | LOX1_5 | linoleate 9S-lipoxygenase | FALSE | -0.011392 | DOWN | -3.6179 | UP | 3.656 |
| Cluster-15808.7404 | -- | lipid transport | FALSE | -1.1026 | DOWN | -1.2113 | FALSE | 0.12441 |
| Cluster-17771.0 | LOX1_5 | linoleate 9S-lipoxygenase | FALSE | 0.22962 | FALSE | -0.85136 | UP | 1.1035 |
| Cluster-15808.10652 | -- | seed biotin-containing protein | FALSE | -1.1106 | DOWN | -1.0732 | FALSE | -0.024394 |
| Cluster-15808.22538 | LOB domain | seed specific protein Bn15D17A | UP | 1.1943 | DOWN | -0.63134 | UP | 1.8502 |
| Cluster-15808.10652 |  | seed biotin containing protein | FALSE | -1.1106 | DOWN | -1.0732 | FALSE | -0.024394 |
| Cluster-17771.1 | LOX1_5 | linoleate 9S-lipoxygenase | FALSE | -0.011392 | DOWN | -3.6179 | UP | 3.656 |
| Cluster-15808.26042 | LOX2S | lipoxygenase | DOWN | -2.5011 | DOWN | -0.51168 | DOWN | -1.9684 |
| **Proline-rich protein and Ferredoxin** | | | | | | | | |
| Cluster-13222.1 | -- | proline-rich protein 4-like | DOWN | -5.8574 | DOWN | -4.3942 | FALSE | -1.4397 |
| Cluster-15655.1 | tuf, TUFM | ferrous iron transport | DOWN | -3.2927 | DOWN | -1.9147 | FALSE | -1.3586 |
| Cluster-12236.0 | petF | ferredoxin | DOWN | -3.1923 | DOWN | -1.8164 | DOWN | -1.3531 |
| Cluster-9783.0 | petH | ferredoxin--NADP+ reductase | DOWN | -3.2501 | DOWN | -1.7486 | DOWN | -1.4834 |
| Cluster-11715.0 | E1.4.7.1 | glutamate synthase (ferredoxin) | DOWN | -2.5564 | DOWN | -2.0139 | FALSE | -0.51722 |
| **Transporter** | | | | | | | | |
| Cluster-15808.7404 | -- | lipid transport | FALSE | -1.1026 | DOWN | -1.2113 | FALSE | 0.12441 |
| Cluster-2223.0 | E3.6.3.8 | Ca2+-transporting ATPase | FALSE | -1.2273 | DOWN | -3.0261 | FALSE | 1.8395 |
| Cluster-15808.11146 | -- | sodium ion transport | FALSE | -0.55066 | DOWN | -0.89384 | FALSE | 0.36163 |
| **Germin like protein** | | | | | | | | |
| Cluster-500.0 | Cupin | Germin-like protein | FALSE | 2.3927 | DOWN | -3.7663 | FALSE | 6.2086 |
| Cluster-500.1 | Cupin | Germin-like protein | FALSE | 3.5649 | DOWN | -2.7507 | UP | 6.3652 |

| Primer name | Gene ID number | Primer | Lenght |
| --- | --- | --- | --- |
